# A Subset of Histone H2B Genes Produces Polyadenylated mRNAs under a Variety of Cellular Conditions

**DOI:** 10.1371/journal.pone.0063745

**Published:** 2013-05-22

**Authors:** Vijayalakshmi Kari, Oleksandra Karpiuk, Bettina Tieg, Malte Kriegs, Ekkehard Dikomey, Heike Krebber, Yvonne Begus-Nahrmann, Steven A. Johnsen

**Affiliations:** 1 Department of Tumor Biology, University Medical Center Hamburg-Eppendorf, Hamburg, Germany; 2 Department of Molecular Oncology, Göttingen Center for Molecular Biosciences, University Medical Center Göttingen, Göttingen, Germany; 3 Department of Molecular Genetics, Institute for Microbiology and Genetics, University Medical Center Göttingen, Göttingen, Germany; 4 Department of Radiation Oncology, University Medical Center Hamburg-Eppendorf, Hamburg, Germany; UMDNJ-New Jersey Medical School, United States of America

## Abstract

Unlike other metazoan mRNAs, replication-dependent histone gene transcripts are not polyadenylated but instead have a conserved stem-loop structure at their 3′ end. Our previous work has shown that under certain conditions replication-dependent histone genes can produce alternative transcripts that are polyadenylated at the 3′ end and, in some cases, spliced. A number of microarray studies examining the expression of polyadenylated mRNAs identified changes in the levels of histone transcripts e.g. during differentiation and tumorigenesis. However, it remains unknown which histone genes produce polyadenylated transcripts and which conditions regulate this process. In the present study we examined the expression and polyadenylation of the human histone H2B gene complement in various cell lines. We demonstrate that H2B genes display a distinct expression pattern that is varies between different cell lines. Further we show that the fraction of polyadenylated *HIST1H2BD* and *HIST1H2AC* transcripts is increased during differentiation of human mesenchymal stem cells (hMSCs) and human fetal osteoblast (hFOB 1.19). Furthermore, we observed an increased fraction of polyadenylated transcripts produced from the histone genes in cells following ionizing radiation. Finally, we show that polyadenylated transcripts are transported to the cytoplasm and found on polyribosomes. Thus, we propose that the production of polyadenylated histone mRNAs from replication-dependent histone genes is a regulated process induced under specific cellular circumstances.

## Introduction

Histones are the major protein component of the eukaryotic chromatin and the transcription of the histone genes is tightly regulated. Histone mRNA levels increase up to 35 fold during the S phase of the cell cycle compared to the G1 phase and back to the basal expression level at the end of the S phase [1]. Unlike the majority of protein-coding mRNAs, replication-dependent histone mRNAs are not spliced and lack polyA tails. Instead their 3′ end contains a highly conserved 16 nucleotide stem-loop sequence and a histone downstream element (HDE) which is recognized by the stem-loop binding protein (SLBP) and U7 snRNPs respectively [2]. In addition to facilitating histone mRNA 3′ end processing, SLBP also facilitates their transport to cytoplasm and stimulates their degradation at the end of the S phase. In some cases, non-replication dependent histone variants such as H3.3, H2A.X and others are expressed throughout the cell cycle, often in a cell type-specific manner, and display the 3′ end polyadenylation instead of a stem loop [3]. Studies from our lab and others have shown that the loss of correct 3′end processing can result in the production of polyadenylated (polyA^+^) histone transcripts from replication-dependent histone genes [4]–[9]. Depletion of various proteins including Cyclin Dependent Kinase 9 (CDK9), RING finger protein 20 (RNF20), RNF40, Nuclear Protein, Ataxia-Telangiectasia Locus (NPAT/p220), Negative Elongation Factor-E (NELF-E), members of the Cap Binding Complex (CBC), or SLBP itself results in the production of polyA^+^ histone transcripts from replication-dependent histone genes [5]–[9]. Importantly, several studies indicate that polyA^+^ histone mRNA levels may increase during various cellular processes including G1 arrest caused by p53 accumulation [8] as well as during differentiation and tumorigenesis [4], [10]–[13]. Finally, up-regulation of polyadenylated histone transcripts can be stimulated by chemical agents such as hydroxyurea (HU) [8].

Despite a number studies reporting the production of polyadenylated histone mRNAs, the functional relevance of these transcripts remains unclear. It remains unknown which of the replication-dependent histone genes can give rise to polyA^+^ transcripts. Furthermore, although it has been reported that polyadenylated histone transcripts produced following SLBP knockdown accumulate in the nucleus [9], it is unclear whether the polyA^+^ histone transcripts produced from the normally replication-dependent genes under normal cellular conditions are exported to the cytoplasm and are actually translated into proteins.

In this study we examined the expression profiles of polyA^+^ and total histone transcripts produced from the entire repertoire of H2B genes and compared these in proliferating and differentiated as well as in primary normal breast and breast cancer tissues. We report that a subset of histone H2B genes also produces polyadenlyated mRNA transcripts. Importantly, we also show that polyadenylated mRNA transcripts of H2B (*HIST1H2BD*) as well as H2A (*HIST1H2AC*) are transported to the cytoplasm where they are also found in the polyribosomal complexes. Importantly, we also show that levels of the polyA^+^ histone transcripts increase during cellular differentiation as well as following the induction of double-strand DNA breaks via gamma-irradiation. Thus, we provide the first evidence that alternative 3′ end processing of histone mRNA transcripts is regulated under specific conditions and that these may lead to functional protein products.

## Materials and Methods

### Cell Culture and Nutlin-3a Treatment

HCT116 cells (human colon cancer cells) were grown in McCoy’s medium containing 10% bovine growth serum (BGS; HyClone, USA) and 1x penicillin–streptomycin (Sigma, St. Louis, USA). Cells were either treated with vehicle (DMSO) or 8 µM of Nutlin-3a (Sigma) for 24 hours and RNA was isolated. H1299 (human non-small cell lung carcinoma cell line), U2OS (human osteosarcoma cell line) and A549 (human alveolar adenocarcinoma cell line) cells were obtained from ATCC and grown in DMEM with high glucose medium containing 10% BGS, sodium pyruvate and 1x penicillin–streptomycin. Tert-immortalized human mesenchymal stem cells (hMSCs) [14] were kindly provided by M. Kassem, Odense University Hospital, Denmark. Cells were cultured in low glucose Minimum Essential Media (MEM) (Life Technologies, Carlsbad, USA) without glutamine and phenol red, supplemented with 10% BGS and 1x antibiotic-antimycotic (Life Technologies). hFOB 1.19 cells were provided by Tom Spelsberg (Mayo Clinic, Rochester, Minnesota) and cultured at the permissive temperature (34 C) in high glucose, phenol red free DMEM/F12 (Invitrogen) supplemented with 10% BGS (Hyclone) and 1X penicillin-streptomycin (Invitrogen). Osteoblast differentiation was induced by shifting to the restrictive temperature (39 C) and growing for 7 days. Adipocyte differentiation of hMSCs was induced as previously described [15] by culturing cells in the presence of 15% BGS, 10 nM dexamethasone (Sigma), 0.45 mM isobutyl-methyl-xanthine (Sigma), 2 µM insulin (Sigma), 10 µM Troglitazone (Sigma) and 1× antibiotic-antimycotic solution. For osteoblast differentiation of hMSCs medium contained 10% BGS, 10 nM dexamethasone, 10 mM β-glycerol phosphate (BGP) (Sigma), 0.2 mM ascorbic acid (Sigma), 10 nM calcitriol (Cayman Chemicals, Ann Arbor, USA) and 1x antibiotic-antimycotic solution.

### Isolation of Total RNA and cDNA Preparation

Total RNA was isolated from cells using TRIzol (Invitrogen) reagent according to the manufacturer’s instructions. Polyadenylated mRNA was isolated from 100 µg of total RNA using the PolyATtract® mRNA Isolation System III (Cat. No. Z5300, Promega, Wisconsin) according to the manufacturer’s instructions. Total or polyA^+^ RNA was reverse transcribed using either random nonamers or polyT primers as indicated in the figure legends. cDNA samples were analyzed by SYBR Green based quantitative real time PCR (qRT-PCR) as described [8]. The expression of individual H2B genes was measured in various cell lines using a linear dilution curve of genomic DNA (gDNA) with known concentrations from a normal, diploid cell line (hMSCs). Finally, relative H2B expression from each gene in different cell lines was normalized to the genomic DNA dilution curve (assuming that each gene is equally represented in a diploid cell) and indicated as “Rel. gDNA units”. The sequences of primers utilized in this study are listed in Supplementary Table S1.

### Cytoplasmic RNA Preparation

For the examination of nuclear and cytoplasmic RNA, HCT116 cells were treated with DMSO or Nutlin-3a for 24 hours and lysed in buffer containing 50 mM Tris HCl (pH 8.0), 140 mM NaCl, 1.5 mM MgCl2, 0.5% v/v Igepal and 1000 U/ml RNase Inhibitor. Cytoplasmic and nuclear fractions were separated by centrifugation at 900 RPM for 10 minutes. RNA was isolated from the cytoplasmic fraction using standard TRIzol extraction method. Unprocessed rRNA and spliced *RPLP0* were used as positive controls for the nuclear and cytoplasmic fractions, respectively (Supplemental Figure S2).

### Polyribosome Purification

Polyribosome purification was carried out essentially as reported with slight modifications [16]. Briefly, HCT116 cells were treated with DMSO or Nutlin-3a for 24 hours and cells were treated with cycloheximide at 37°C for 30 min brought to the final concentration of 100 µg/ml. Cell lysates were prepared in lysis buffer containing 20 mM HEPES (pH 7.5), 125 mM KCl, 5 mM MgCl_2_, 2 mM DTT, 0.5% NP-40, 100 µg/ml of cycloheximide and 100 U/ml of RNase inhibitor along with protease inhibitors. Cleared lysates were loaded on to the sucrose gradient 8–50% in lysis buffer and centrifuged at 34,000 RPM for 130 minutes. Fractions were collected from the gradients and RNA was extracted from polyribosome fractions.

## Results

### Expression of Replication-dependent Histone H2B Gene Transcripts in Different Cell Lines

**Figure 1:**
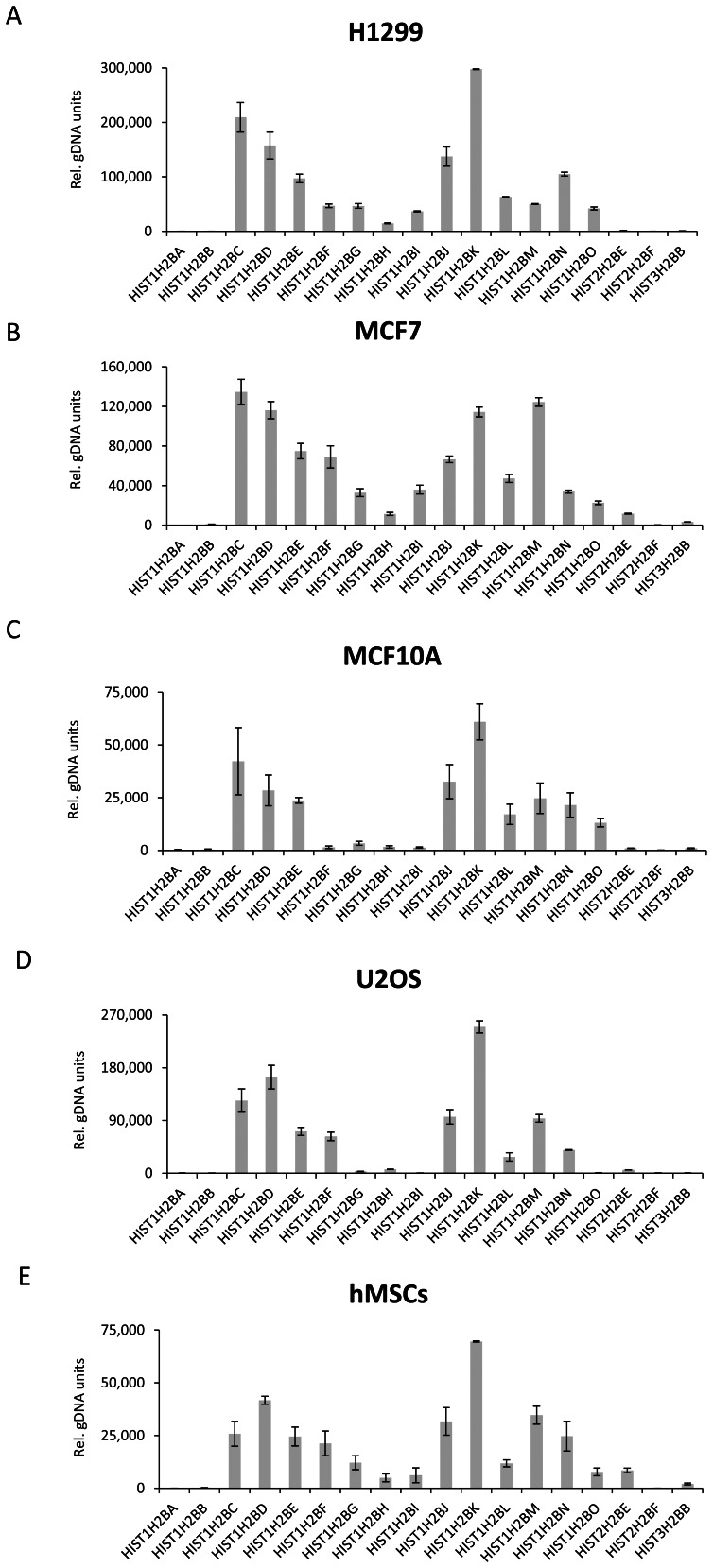
Expression of the histone H2B gene complement in different cell lines. Expression of different H2B genes in the indicated cell lines was analyzed by qRT-PCR. Relative expression values between the individual genes were normalized using diploid genomic DNA (see materials and methods) and indicated as “Rel. gDNA units”. Mean±SD, n = 3.

The metazoan core histone genes are clustered together in the genome. In mammals, there are two major histone gene clusters on chromosome 6p21–p22(*HIST1*) and 1q21 (*HIST2*) as well as one minor cluster on 1q42 (*HIST3*) [17]. Each of the histone proteins is encoded by several histone genes and there are 18 histone H2B genes reported for human. To date it is unclear to what extent each of the individual histone genes are actually expressed, and whether this expression varies between tissues, cell types or under different physiological conditions. Since the expression levels of the various H2B genes remain largely unknown, we examined the expression levels of replication-dependent H2B transcripts in different cell lines including H1299, MCF7, MCF10A, U2OS and hMSCs via real time quantitative PCR (qRT-PCR) (Fig. 1 A–E). The expression of individual H2B gene transcripts was represented as relative genomic DNA units (Rel. gDNA) as described in materials and methods to enable the quantitative comparison between different genes. For each cell line tested we observed distinct H2B gene expression profiles. While many genes were either consistently expressed at medium to high levels (*HIST1H2BC*, *HIST1H2BD, HIST1H2BE, HIST1H2BJ, HIST1H2BK, HIST1H2BL, HIST1H2BM and HIST1H2BN*) and others were very low or undetectable in all cell lines tested (*HIST1H2BA*, *HIST1H2BB*, *HIST2H2BF* and *HIST2H2BB*) other genes displayed cell line-specific expression. For example, while *HIST1H2BM* is expressed at medium levels in H1299, MCF10A, U2OS and hMSC cells, it represents one of the major expressed H2B genes in MCF7 cells. Similarly, while *HIST2H2BE* expression was nearly undetectable in H1299, MCF10A and U2OS cell lines, moderate expression was observed in MCF7 and hMSCs. The *HIST1H2BG* and *HIST1H2BI* genes also showed cell line-specific expression in which they were moderately expressed in H1299, MCF7 and hMSCs, but very low in U2OS and MCF10A cells. HIST1H2BF was also broadly expressed in the cell lines except in MCF10A where expression was very low. Thus, the repertoire of H2B genes expressed appears to be regulated in a cell context-specific manner.

### H2B mRNAs are Differentially Polyadenylated Upon p53-induced Cell Cycle Arrest

**Figure 2:**
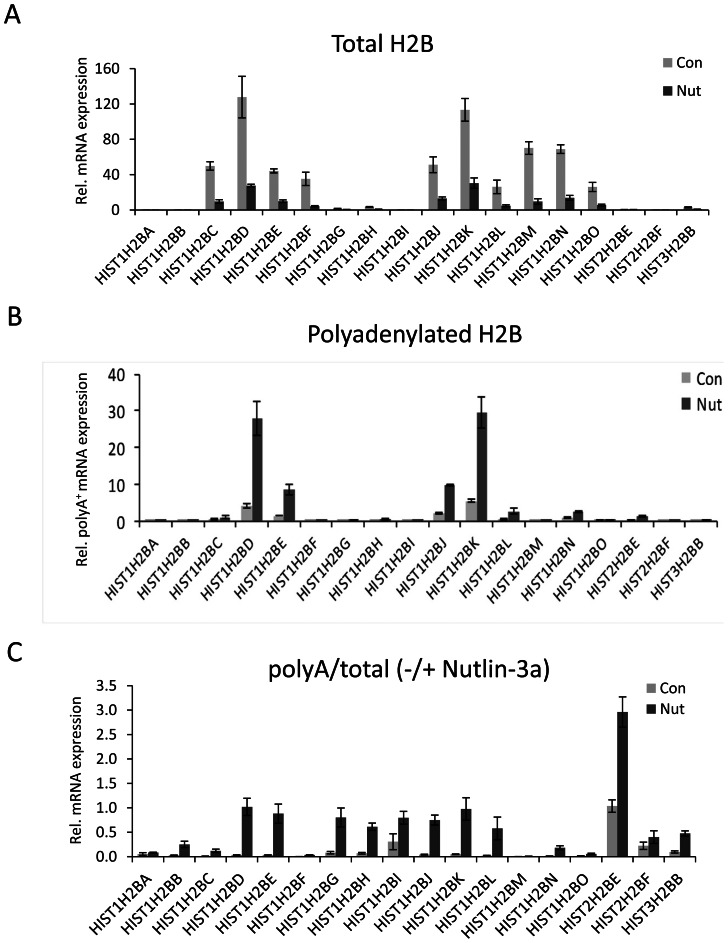
Nutlin-3a treatment down-regulates the expression of normal replication-dependent histone H2B genes and up-regulates the expression polyA^+^ transcripts. (A) Total expression of different replication-dependent histone H2B genes in control and Nutlin-3a treated HCT116 cells. Total RNA was reverse transcribed using random primers and analyzed by qRT-PCR for H2B genes as in Fig. 1. Mean±SD, n = 3. (B) Expression of polyA^+^ histone H2B transcripts in HCT116 cells upon Nutlin-3a treatment. PolyA^+^ mRNA was purified as described in materials and methods and reverse transcribed using random primers. Transcript levels of polyA^+^ H2B genes were analyzed by qRT-PCR. Mean±SD, n = 3. (C) Levels of polyA^+^ H2B transcripts normalized to the total H2B levels from (B). Mean±SD, n = 3.

In our previous studies, we demonstrated that the manipulation of epigenetic regulatory pathways [6], [7] or the induction of a G1 cell cycle arrest [8] results in an increase in the production of spliced and polyadenylated transcripts from the *HIST1H2BD* and *HIST1H2AC* genes. Thus, after comparing the overall expression levels of different histone genes in various cell lines, we next examined which of them give rise to polyA^+^ transcripts. In order to do this, we purified total and polyA^+^ mRNA from HCT116 cells treated for 24 hours with Nutlin-3a, a small molecule inhibitor of the p53 ubiquitin ligase MDM2 [18] which induces a G1 cell cycle arrest in p53-proficient HCT116 cells [8], and examined the expression of each of the H2B genes via qRT-PCR analysis. To validate the purity of polyA^+^ mRNA purified from control and Nutlin-3a treated cells, we analyzed for the presence of ribosomal rRNA transcripts (5.8S rRNA and 18S rRNA) which are not polyadenylated (Fig. S1A). Consistent with the earlier reports Nutlin-3a treatment decreased the overall expression of all detectable H2B transcripts (irrespective of polyadenylation status) (Fig. 2A). Interestingly, many H2B genes demonstrated a significant increase in the amount of polyA^+^ transcript production following Nutlin-3a treatment (Fig. 2B). Notably, the *HIST1H2BD*, *HIST1H2BE*, *HIST1H2BJ*, *HIST1H2BK* genes were highly expressed and also showed a significant increase in the fraction of polyadenylated transcripts (Fig. 2B, 2C). Normalization of polyA^+^ H2B mRNA levels to total H2B expression revealed that the fraction of polyadenylated transcripts is similarly up-regulated upon Nutlin-3a treatment for several genes irrespective of their overall expression levels. For example, the levels of polyadenylated transcripts from the *HIST1H2BG*, *HIST1H2BH*, and *HIST1H2BI* genes are upregulated to a similar extent as the more highly expressed genes *HIST1H2BD* and *HIST1H2BK* (Fig. 2C). Importantly, not all transcribed H2B genes demonstrated these effects. For example, HIST1H2BC, HSIT1H2BF, HIST1H2BM and HIST1H2BO are all expressed at significant levels, but show only very little or no evidence of polyA^+^ transcripts (Fig. 2). Thus the production of polyadenylated mRNAs from histone H2B genes is regulated in a gene-specific manner.

**Figure 3:**
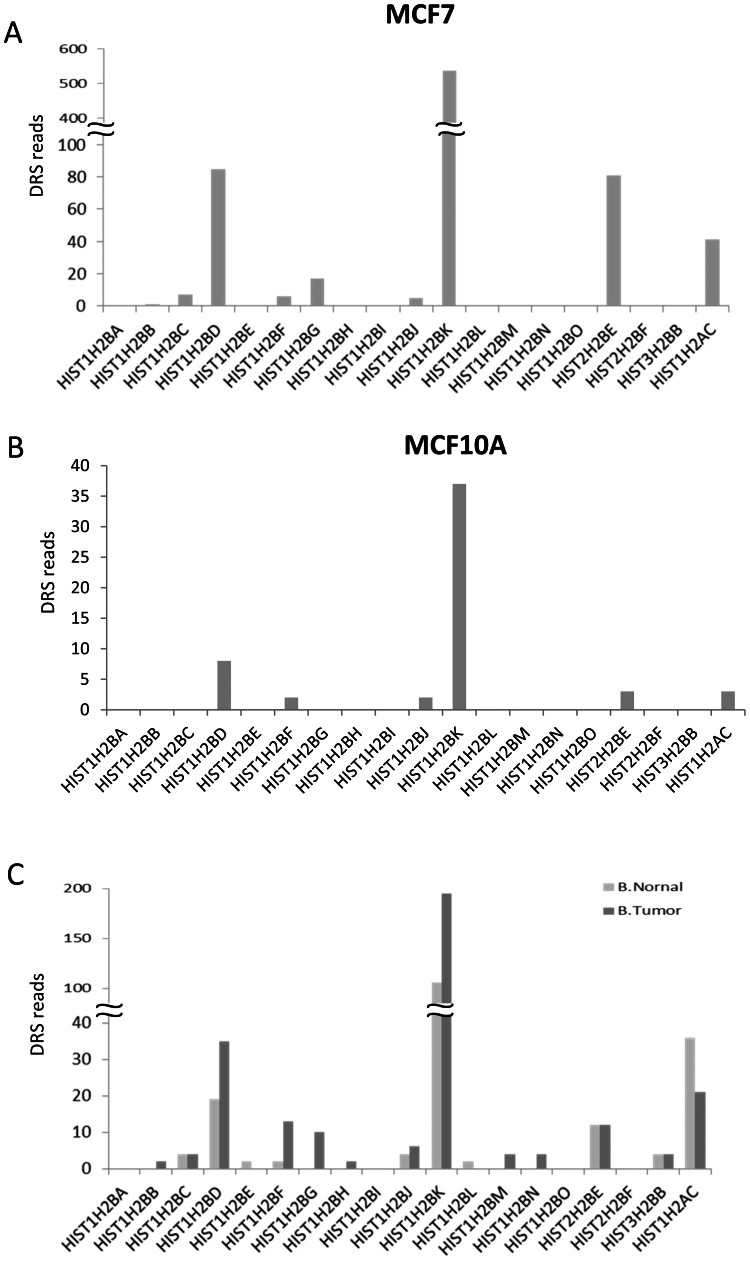
Polyadenylation of histone H2B genes assigned using polyadenylation and alternative polyadenylation (APA) map. Number of reads at polyA sites on different replication- dependent histone H2B genes which are mapped using xPAD server (http://johnlab.org/xpad/) in (A) MCF7 (human breast cancer cell line), (B) MCF10A (immortalized human mammary epithelial cell line) cells and (C) normal and breast tumor tissues.

Recent studies using a transcriptome-wide direct RNA sequencing (DRS) approach enable the precise mapping and quantification of polyadenylation sites as well as the identification of differentially polyadenylated RNA transcripts [19], [20]. We used the recently developed xPAD server genome browser (http://johnlab.org/xpad/) to map the DRS reads on histone H2B genes for the breast cancer cell line MCF7 (Fig. 3A), as well as the normal mammary epithelial cell line MCF10A (Fig. 3B). Consistent with the data in HCT116 cells, the mapping of DRS reads demonstrated that only a subset of H2B genes has detectable polyadenylation sites in MCF7 and MCF10A cells (Fig. 3, Supplementary Fig. S3). Moreover, H2B transcripts identified as being highly polyadenylated in HCT116 cells (e.g., *HIST1H2BD* and *HIST1H2BK*; Fig. 2B) were also found to possess polyadenylation sites in MCF7 and MCF10A cells. Interestingly, using a further set of DRS mapping data, we observed that the number of reads identified for polyA^+^ histone transcripts increased in tumor breast samples compared to normal breast epithelium (Fig. 3C) possibly suggesting that increased levels of polyA^+^ histone transcripts may provide an advantage to tumor cells.

### Polyadenylated Histone H2B Transcripts are Transported to the Cytoplasm and Found on Polyribosomes

**Figure 4:**
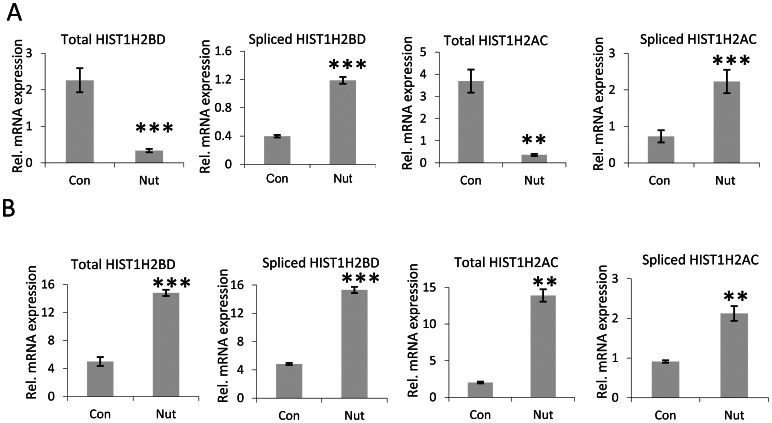
Expression of normal and PolyA^+^ *HIST1H2BD* and *HIST1H2AC* transcripts in HCT116 cells. (A) Cells were treated with Nutlin-3a as in Fig. 2. RNA was reverse transcribed into cDNA using both random and poly-T primers to check the mRNA levels of *HIST1H2BD* and *HIST1H2AC*, total and polyadenylated transcripts respectively. Values were normalized to *RPLP0* expression. Mean±SD, n = 3. (B) Enrichment for polyadenylated histone transcripts using PolyATtract® mRNA Isolation System III. Total RNA was used to isolate polyadenylated RNA and reverse transcribed using poly-T primers. Expression of total and polyA^+^ *HIST1H2BD* and *HIST1H2AC* transcripts was analyzed by qRT-PCR. Values were normalized to *RPLP0* expression. Mean±SD, n = 3. P-values were calculated and statistical significance was represented as follows (**P≤0.01; ***P≤0.001).

Metazoan replication-dependent histone mRNAs are single exonic and are not spliced. Importantly, the inclusion of an intron prevents proper stem loop-dependent mRNA 3′ end processing suggesting, that stem loop-directed 3′ end processing of histone mRNAs is mutually exclusive with splicing and polyadenylation [21]. We have previously shown that some histone genes (e.g. *HIST1H2BD* and *HIST1H2AC*) produce both canonically processed replication-dependent mRNAs as well as longer, spliced replication-independent mRNAs produced using a downstream second exon [6], [8]. Due to the size of the primary transcript and the distance between the canonical 3′ end processing site and the polyadenylation site, these two transcripts can more easily be distinguished from their non-polyadenylated counterparts than transcripts produced from polyadenylation sites located immediately downstream of the canonical stem loop-directed 3′ end processing site (e.g., *HIST1H2AA*). Thus, we verified the expression of total and polyadenylated *HIST1H2BD* and *HIST1H2AC* transcripts in HCT116 cells arrested in G1 phase by Nutlin-3a treatment (Fig. 4A). Consistent with our previous results, Nutlin-3a treatment increased the levels of polyA^+^ *HIST1H2BD* and *HIST1H2AC* transcripts while decreasing their overall levels (i.e., canonically processed and polyadenylated together). These results were further verified in polyA^+^ purified mRNA (Fig. 4B).

**Figure 5:**
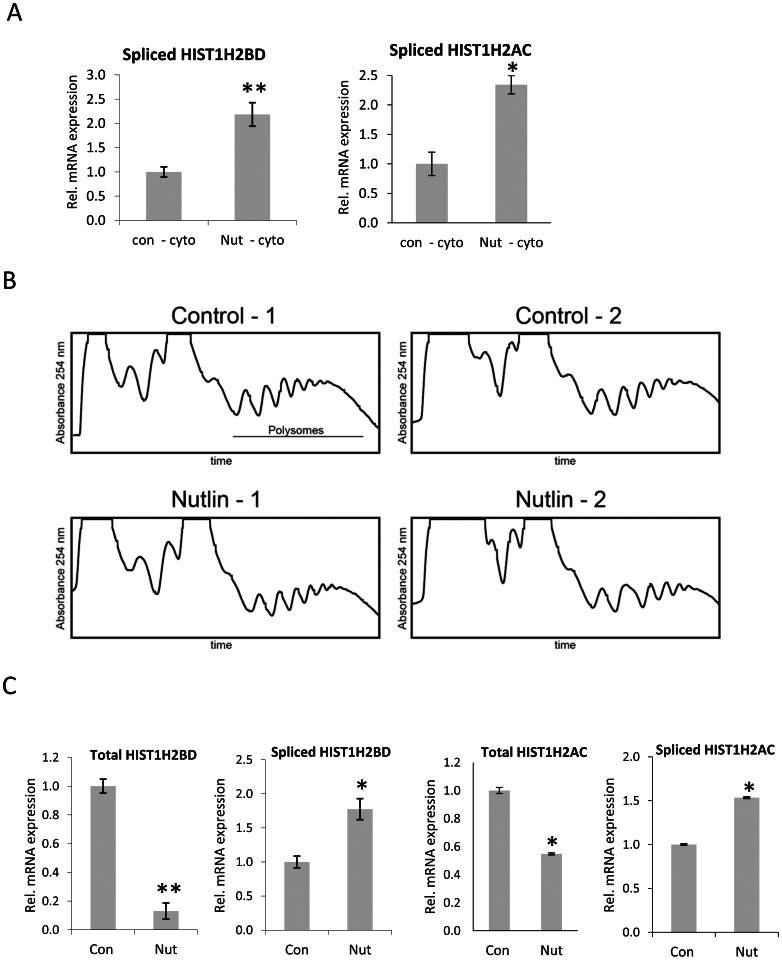
Spliced *HIST1H2BD* and *HIST1H2AC* transcripts are transported to the cytoplasm and found on polyribosomes. (A) Polyadenylated *HIST1H2BD* and *HIST1H2AC* mRNA is transported to the cytoplasm. Cytoplasmic RNA was isolated from HCT116 cells treated either with DMSO or Nutlin-3a for 24 hours. RNA was reverse transcribed using random primers and analyzed for spliced *HIST1H2BD* and *HIST1H2AC* transcript by qRT-PCR. Values were normalized to 18S rRNA. Mean±SD, n = 3. (B) Representative polyribosome profiles obtained after sucrose gradient fractionation from DMSO and Nutlin-3a treated HCT116 cells. The x-axis represents the time of elution and y-axis represents the absorbance at 254 nm, indicating the RNA content. Polysome profiles were indicated in the figure. (C) RNA was extracted from the indicated polyribosome fractions of DMSO and Nutlin-3a treated cells and reverse transcribed using random primers. Expression of total and spliced *HIST1H2BD* and *HIST1H2AC* mRNA was analyzed by qRT-PCR and values were normalized to 18S rRNA. Mean±SD, n = 2. P-values were calculated and statistical significance is represented as follows (*P≤0.05; **P≤0.01).

Although a number studies examined the “expression” of polyA^+^ histone transcripts (primarily through microarray analysis), whether or not these transcripts are actually exported from the nucleus and translated was unclear. We hypothesized that polyA^+^ histone mRNAs may be translated and give rise to proteins. To examine whether polyA^+^ histone mRNA is transported to the cytoplasm we isolated cytoplasmic RNA from HCT116 cells and examined it for the presence of spliced and polyadenylated histone *HIST1H2BD* and *HIST1H2AC* mRNA. qRT-PCR analysis with the cytoplasmic RNA confirmed the presence of *HIST1H2BD* and *HIST1H2AC* spliced transcripts, indicating that polyA^+^ histone mRNAs are indeed transported to the cytoplasm (Fig. 5A). To further determine whether these polyA^+^ histone mRNA transcripts are actually translated, we isolated polyribosomes from control and Nutlin-3a treated HCT116 cells. The representative polyribosome profiles are shown in Fig. 5B. qRT-PCR analyses of polyribosome-bound RNA clearly demonstrated the presence of polyA^+^ histone gene transcripts, supporting the conclusion that polyA^+^ transcripts may give rise to proteins and thereby contribute to the maintenance of histone protein levels. Furthermore, we also observed a Nutlin-3a-induced increase of the polyribosome-bound polyA^+^ fraction vs. a decrease in polyribosome-bound total histone mRNA levels (Fig. 5C).

### Radiation-induced Expression of polyA^+^ Gene Transcripts

**Figure 6:**
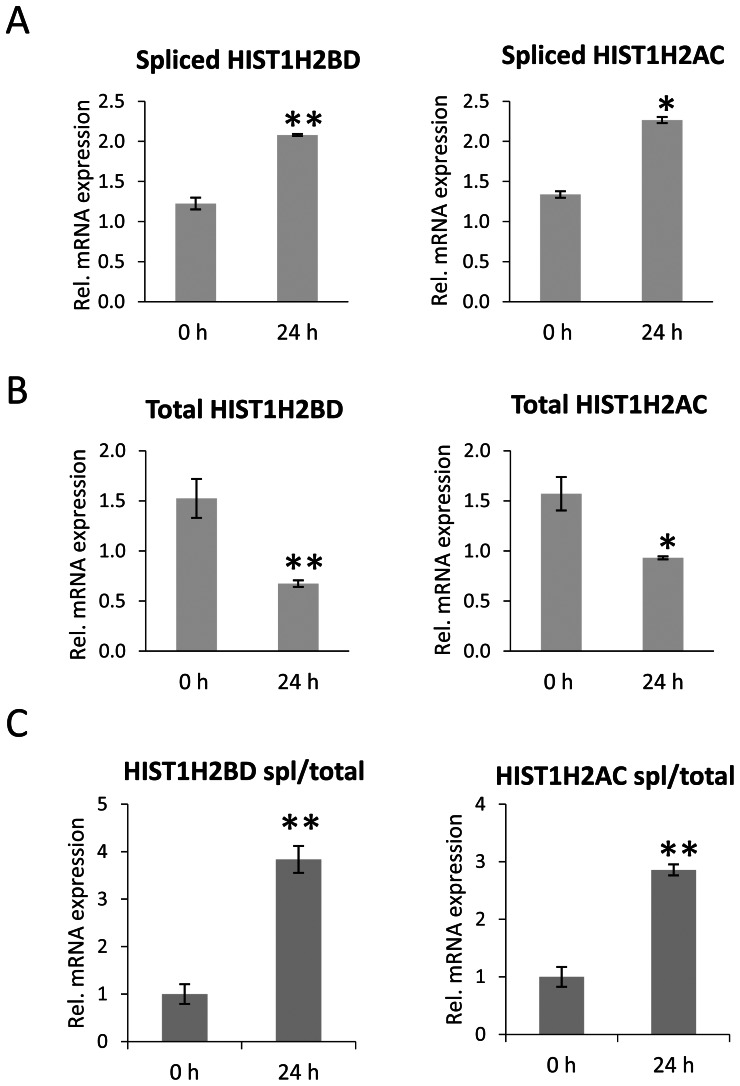
Radiation induced elevated expression of spliced histone transcripts. A549 cells were exposed to gamma-irradiation (6 Gy) and incubated for 24 hours. RNA was extracted and analyzed by qRT-PCR for (A) spliced and (B) total *HIST1H2BD* and *HIS1H2AC*. Values were normalized to *RPLP0*. Mean±SD, n = 3. (C) Expression of spliced *HIST1H2BD* and *HIST1H2AC* transcripts was normalized to the total *HIST1H2BD* and *HIST1H2AC* levels. P-values were calculated and statistical significance is represented as follows (*P≤0.05; **P≤0.01).

After establishing that polyA^+^ transcripts can be transported to cytoplasm and translated, we investigated whether the levels of polyA^+^ histone transcripts may be regulated under physiological circumstances. Initially, we tested whether exposure of A549 lung carcinoma cells to γ-radiation (6 Gy) affects the levels of polyA^+^ histone mRNAs. Consistent with the effects of Nutlin-3a-induced cell cycle arrest, 24 h after irradiation the mRNA levels of spliced *HIST1H2BD* and *HIST1H2AC* (Fig. 6A) were significant elevated despite an overall decrease in total histone transcript levels (Fig. 6B).

### PolyA^+^ Histone mRNA Expression is Up-regulated during Cellular Differentiation

**Figure 7:**
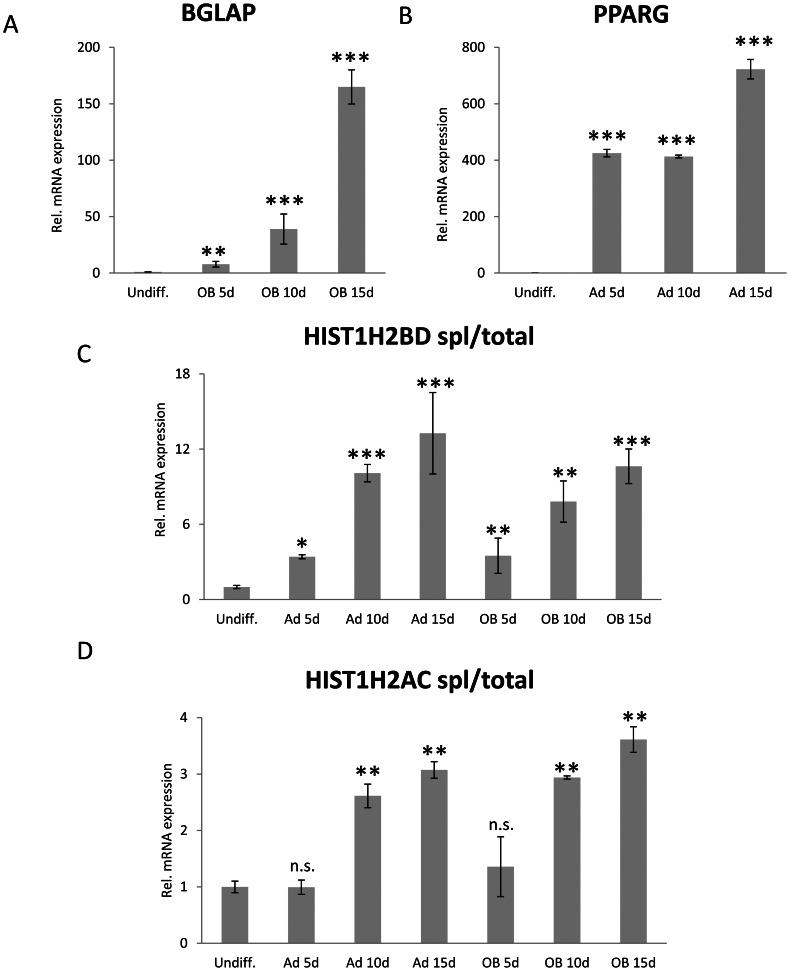
Differentiation of uncommitted mesenchymal stem cells results in elevated expression of spliced up to histone transcripts. (A, B) hMSCs were differentiated into (A) adipocytes or (B) osteoblasts for 15 days. Expression of marker genes *PPARG* for adipocytes and *BGLAP* for osteoblasts was analyzed by qRT-PCR. Values were normalized to *HNRNPK* expression. Mean±SD, n = 3. (C, D) The expression of (C) spliced *HIST1H2BD* or (D) spliced *HIST1H2AC* was analyzed by qRT-PCR using same samples as in (A) and (B). To obtain relative amounts of spliced transcript its expression was normalized to (C) total *HIST1H2BD* or (D) *HIST1H2AC* expression. Mean±SD, n = 3. P-values were calculated and statistical significance is represented as follows (ns P>0.05; *P≤0.05; **P≤0.01; ***P≤0.001).

**Figure 8:**
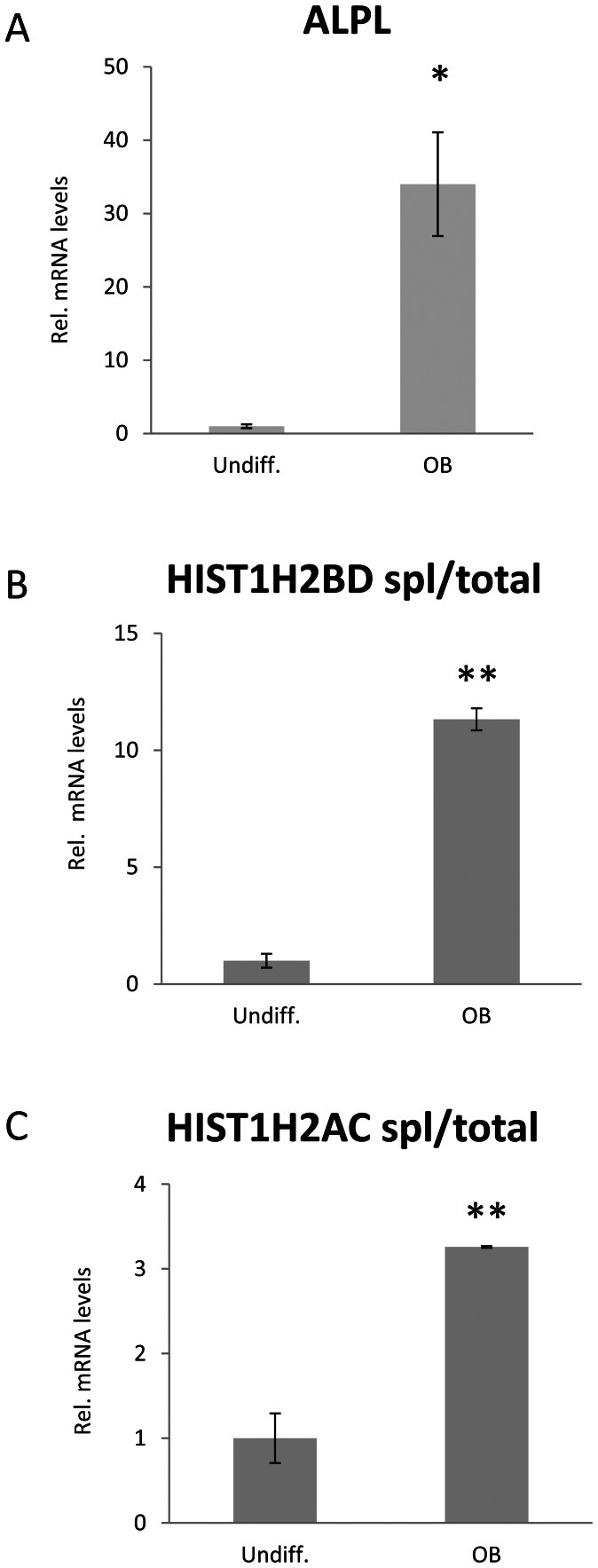
Expression of spliced histone transcripts are increased during committed osteoblast differentiation. (A) hFOB 1.19 cells were differentiated into osteoblasts for 7 days. Alkaline phosphatase (*ALPL*) expression in undifferentiated (undiff.) and differentiated (OB) cells was analyzed by qRT-PCR. Values were normalized to *HNRNPK* expression. Mean±SD, n = 3. (B, C) Samples shown in (A) were examined for (B) spliced *HIST1H2BD* and (C) *HIST1H2AC* expression. Values were normalized to total *HISTH2BD* and *HISTH2AC* respectively. Mean±SD, n = 3. P-values were calculated and statistical significance is represented as follows (*P≤0.05; **P≤0.01).

We previously hypothesized that terminal cellular differentiation may result in changes in histone mRNA polyadenylation [8]. To test this hypothesis, we utilized an immortalized human mesenchymal stem cell (hMSC) line which can be differentiated to the osteoblast, adipocyte or chondrocyte lineages [14]. We differentiated hMSCs into either adipocytes or osteoblasts for 5, 10 or 15 days and confirmed the expression of differentiation-specific genes *PPARG* (Fig. 7A) for the adipocyte lineage and *BGLAP* for the osteoblast lineage (Fig. 7B) before analyzing the expression of spliced *HIST1H2BD* and *HIST1H2AC* transcripts (Fig. 7C, D). Consistent with our hypothesis, the expression of spliced *HIST1H2BD* (Fig. 7C) and *HIST1H2AC* (Fig. 7D) mRNAs was significantly increased in differentiated adipocytes and osteoblasts compared to undifferentiated hMSCs irrespective of the differentiation lineage. To further investigate whether the observed effect of differentiation on histone mRNA polyadenylation could be recapitulated in another differentiation model, we also differentiated human fetal osteoblasts (hFOB 1.19) [22] for 7 days. Induction of *ALPL* expression confirmed successful differentiation of hFOB 1.19 cells into the osteoblast lineage (Fig. 8A). Importantly, consistent with the effects observed during hMSC differentiation, the fraction of polyadenylated and spliced *HIST1H2BD* (Fig. 8B) and *HIST1H2AC* (Fig. 8C) was significantly increased upon differentiation into the osteoblast lineage (Fig. 8B, C). Together these results suggest that the expression of polyadenylated histone mRNAs increases in various differentiation pathways and models.

## Discussion

In proliferating metazoan cells most histone synthesis is coupled to DNA replication and occurs during the S phase of the cell cycle. While the transcription of replication-dependent histone mRNAs is cell cycle regulated, the histone mRNAs themselves also have several specific features. First of all, they are not polyadenylated at their 3′ ends, but instead possess a specific stem-loop structure that is recognized by a unique 3′ end processing machinery and aids both in the 3′ end cleavage as well as in nuclear export and translation. Secondly, replication-dependent histone transcripts contain only one exon and, unlike most mRNAs, are not spliced. Despite significant transcriptional regulation during S phase, a certain degree of basal level histone synthesis was also observed throughout the cell cycle, independently of replication [23]. Since stem-loop processing is coupled to the S phase, this phenomenon may be explained by mRNA processing events independent of the cell cycle. This hypothesis was supported by the identification of replication-independent histone mRNAs that are produced from the same histone genes as replication-dependent transcripts, but additionally contain a polyadenylation site downstream of their stem loop sequence [4], [8]. Interestingly, polyA^+^ histone transcripts from replication-dependent histone genes were also detected in *C. elegans* [24] as well as in mouse ES cells and post mitotic neurons [25] via direct RNA sequencing, suggesting that polyA^+^ transcripts may have emerged early during evolution to facilitate the basal histone production.

Increased production of polyA^+^ histone transcripts was shown to be induced by a wide range of factors including depletion of epigenetic regulators, induction of DNA damage or serum starvation [6]–[8]. Furthermore, numerous microarray-based studies have observed changes in the “expression” of replication-dependent histone mRNA transcripts during tumorigenesis and differentiation [12], [26]. Given the fact that these studies were based on poly-T reverse transcription, it seems likely that histone mRNA polyadenylation is a process regulated under diverse conditions. Tumor suppressor p53 mediated cell-cycle arrest, implicated in the regulation of proliferation and tumorigenesis, also controls the expression of polyA^+^ histone transcripts via p21-dependent cell cycle arrest [8]. Despite numerous studies reporting the expression of polyA^+^ histone mRNAs from the replication-dependent histones genes, the functional importance of these transcripts remains unknown and has been refuted [9]. In this study we examined the expression of polyA^+^ histone transcripts from replication-dependent histone genes which normally primarily produce 3′stem-loop containing mRNAs, under various cellular conditions.

**Figure 9:**
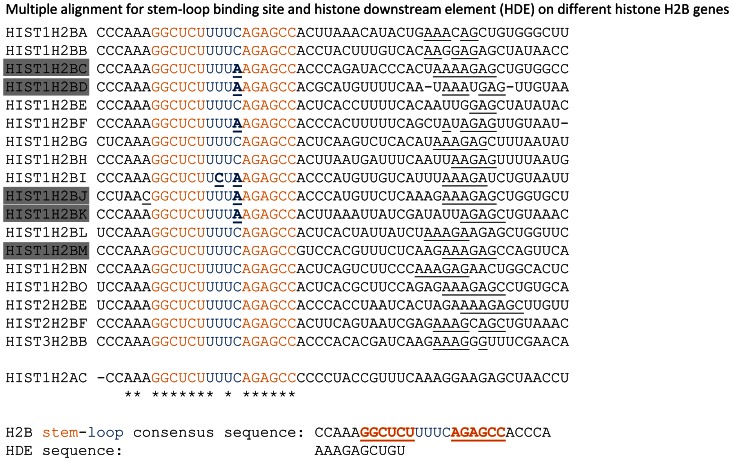
Comparison of stem-loop sequences in H2B genes. Alignment of the stem-loop sequences of H2B genes performed with ClustalW2 multiple sequence alignments tools (http://www.ebi.ac.uk/Tools/msa/clustalw2/). Highly expressed histones are marked in grey. Stem loop sequence is shown in orange, bases that are different from canonical are underlined.

First of all, using the histone H2B gene complement as a model system for our studies, we compared the expression of different H2B genes. Surprisingly, we observed a wide range of expression of the individual genes, suggesting that the regulation of histone transcription and mRNA processing is gene-specific and more complex process than may have been previously assumed. Moreover, we demonstrated that different cell lines exhibit distinct expression patterns of total and polyadenylated H2B mRNA. The efficiency of transcription might be dependent on promoter context and/or mRNA 3′ UTR sequence. A pervious study demonstrated that minor changes in the stem loop sequence can significantly affect 3′ end processing efficiency [27]. Interestingly, a recently published study shows that the structure, rather than the sequence of the stem loop is essential for proper SLBP binding [28]. Furthermore, the human 3′ exonuclease (3′hExo) involved in trimming of histone mRNAs cleaved in an SLBP-directed manner binds to specific sites within the 3′ stem loop including C15 within the loop sequence. Surprisingly, a number of H2B mRNAs depart from the stem loop consensus sequence (Fig. 9). Interestingly, we observed that 4 out of 5 highly expressed H2B histone genes (*HIST1H2BC*, *HIST1H2BD*, *HIST1H2BJ* and *HIST1H2BK*) possess a single nucleotide mutation at C15 (C to A) within the loop sequence (Fig. 9). In addition, the 5 nucleotides 5′ to the stem loop (CCAAA) are also recognized by SLBP [28]. Interestingly, the *HIST1H2BJ* RNA departs from the consensus 3′ end processing sequence and has a C at position -1 relative to the stem loop sequence. Consistent with its consensus stem loop composition, the highly expressed H2B gene, *HIST1H2BM*, was not found to be significantly polyadenylated in our studies in HCT116 cells or in DRS data for MCF7 or MCF10A cells. Thus, it appears likely that a canonical stem loop sequence promotes efficient histone mRNA 3′ end processing *in vivo* while single nucleotide changes in the loop or 5′ sequences may be sufficient to allow for alternative mRNA 3′ end processing via polyadenylation.

In addition to expanding upon our previous observations that polyA^+^ transcripts are produced from H2B genes, we have demonstrated that not all the H2B genes give rise to polyA^+^ mRNAs. While the expression levels of the individual H2B genes frequently correlate with the amount of polyA^+^ transcripts produced, additional factors, including the composition of the 3′ stem loop sequence appear to influence the mode and efficiency of 3′ end processing. Given our recent findings that NPAT not only supports transcriptional regulation of histone genes, but also promotes proper 3′ end processing [8], it is likely that sequences within the proximal promoter regions of the histone genes may also promote 3′ end processing.

Whether or not polyA^+^ histone mRNAs play a physiological role remains unclear. However, since polyA^+^ transcripts have longer half-lives compared to their S phase counterparts [4], their expression may by necessary to compensate for decreased histone synthesis, for example in non-proliferating, terminally differentiated cells. Indeed, our results demonstrate that the levels of polyadenylated histone mRNAs significantly increase during cellular differentiation. Since in mRNA levels may not necessarily result in the production of a functional protein, we also performed polyribosome purification in cell cycle arrested cells and demonstrated for the first time that polyA^+^ transcripts are indeed polyribosomal and therefore likely give rise to functional histone proteins. However, whether the translated histone proteins produced from these transcripts are indeed incorporated into chromatin remains to be elucidated.

Since p53 accumulation following Nutlin-3a treatment mainly results in a prominent cell cycle arrest, we hypothesized that polyA^+^ transcript production is generally activated upon cell cycle arrest, when replication-dependent histone synthesis in not possible [8]. Moreover, we further hypothesized that conditions such as double-strand DNA break repair, which require massive changes in chromatin structure and histone exchange [29], may be particularly dependent upon polyA^+^ histone transcripts for the generation of new histone proteins. In support of this hypothesis we observed an up-regulation of polyA^+^ transcripts following γ-radiation. Based on these findings, we propose that induction of polyA^+^ histone transcripts may be a general mechanism to overcome a deficit in replication-dependent histone transcripts cause by cell cycle alterations. These transcripts may be essential for maintaining proper DNA packing and chromatin in the absence of replication where there is no expression of replication-dependent histone genes. Further studies are required to investigate the role of polyA^+^ histone transcripts in cells or tissues like neurons or cardiomyocytes, which are terminally differentiated and no longer divide. Such studies will require the further elucidation of which genes encoding the other core histones are expressed and which of these are polyadenylated. In conclusion, our data demonstrate that production of polyA^+^ histone transcripts is subject to specific regulation and becomes induced during differentiation, DNA damage or cell cycle arrest most likely in order to maintain histone protein levels.

## Supporting Information

Figure S1 **(Related to** **Figure 2** **).** (A) Quality of polyA^+^ mRNA purified using PolyA Ttract® mRNA Isolation System III. To analyze the relative enrichment of polyA^+^ RNA, 100 ng of total and polyA^+^ RNA from control and Nutlin-3a treated cells was reverse transcribed using random nonamers and analyzed for 5.8S and 18S rRNA transcripts by qRT-PCR. (B) qRT-PCR analysis for *HNRNPK* mRNA expression in polyA^+^ purified mRNA from control and Nutlin-3a treated cells
(PDF)

Figure S2 **(Related to** **Figure 5** **).** Purity of cytoplasmic and nuclear RNA. To check the purity of cytoplasmic and nuclear fractions RNA was analyzed by qRT-PCR for (A) un-spliced 5.8 S rRNA (specific for nuclear), (B) *RFLP0* (cytoplasmic) from control and Nutlin-3a treated cells.
(PDF)

Figure S3 **UCSC genome browser views of DRS data depicted in** **Fig. 3** **.**
(PDF)

Table S1 **Primers used in this study.**
(DOCX)
