# Supplementary figures and images for: A Subset of Histone H2B Genes Produces Polyadenylated mRNAs under a Variety of Cellular Conditions
Source: PLoS One. 2013 May 22;8(5):e63745. doi: 10.1371/journal.pone.0063745 (PMC3661734; doi:10.1371/journal.pone.0063745)

Supplementary Figure S1

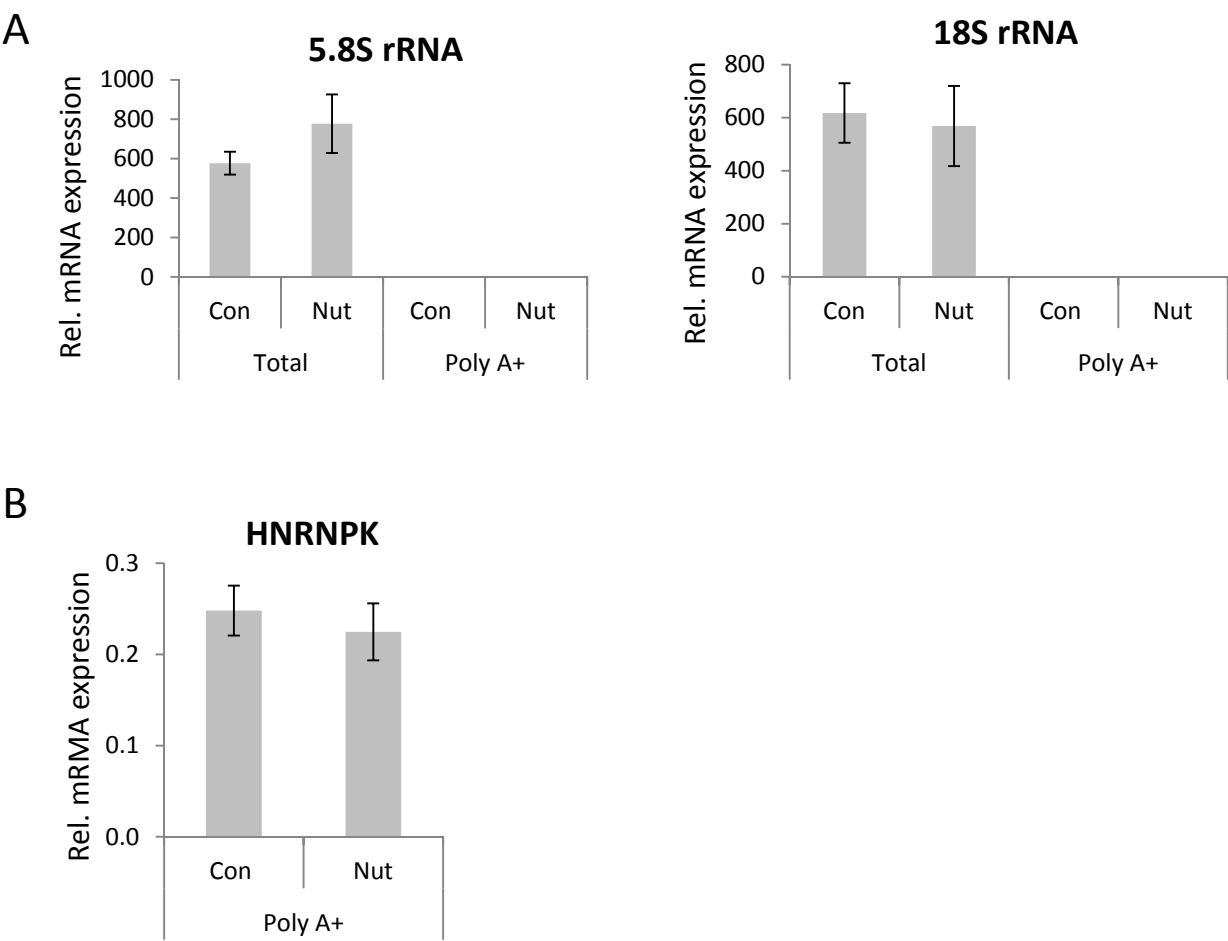

Supplement: Figure S1 — (Related to Figure 2 ). (A) Quality of polyA+ mRNA purified using PolyA Ttract® mRNA Isolation System III. To analyze the relative enrichment of polyA+ RNA, 100 ng of total and polyA+ RNA from control and Nutlin-3a treated cells was reverse transcribed using random nonamers and analyzed for 5.8S and 18S rRNA transcripts by qRT-PCR. (B) qRT-PCR analysis for HNRNPK mRNA expression in polyA+ purified mRNA from control and Nutlin-3a treated cells (PDF) [file pone.0063745.s001.pdf]

# Supplementary Figure S2

A

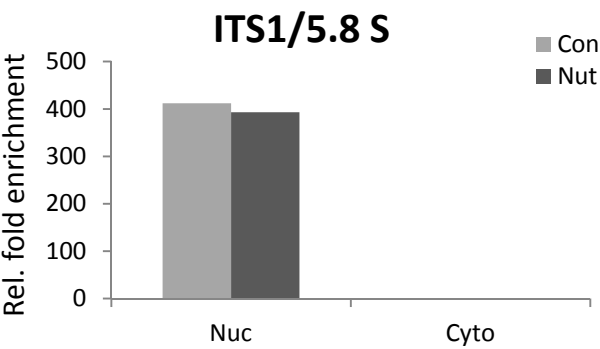

B

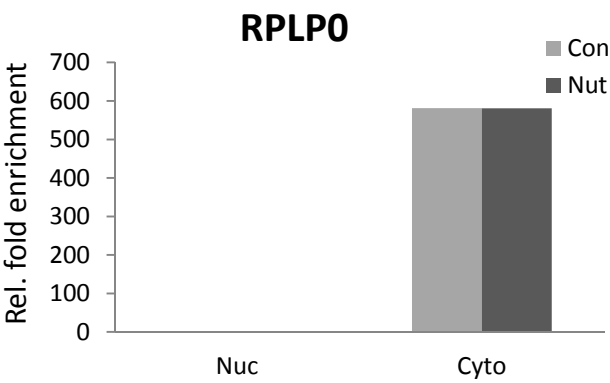

Supplement: Figure S2 — (Related to Figure 5 ). Purity of cytoplasmic and nuclear RNA. To check the purity of cytoplasmic and nuclear fractions RNA was analyzed by qRT-PCR for (A) un-spliced 5.8 S rRNA (specific for nuclear), (B) RFLP0 (cytoplasmic) from control and Nutlin-3a treated cells. (PDF) [file pone.0063745.s002.pdf]

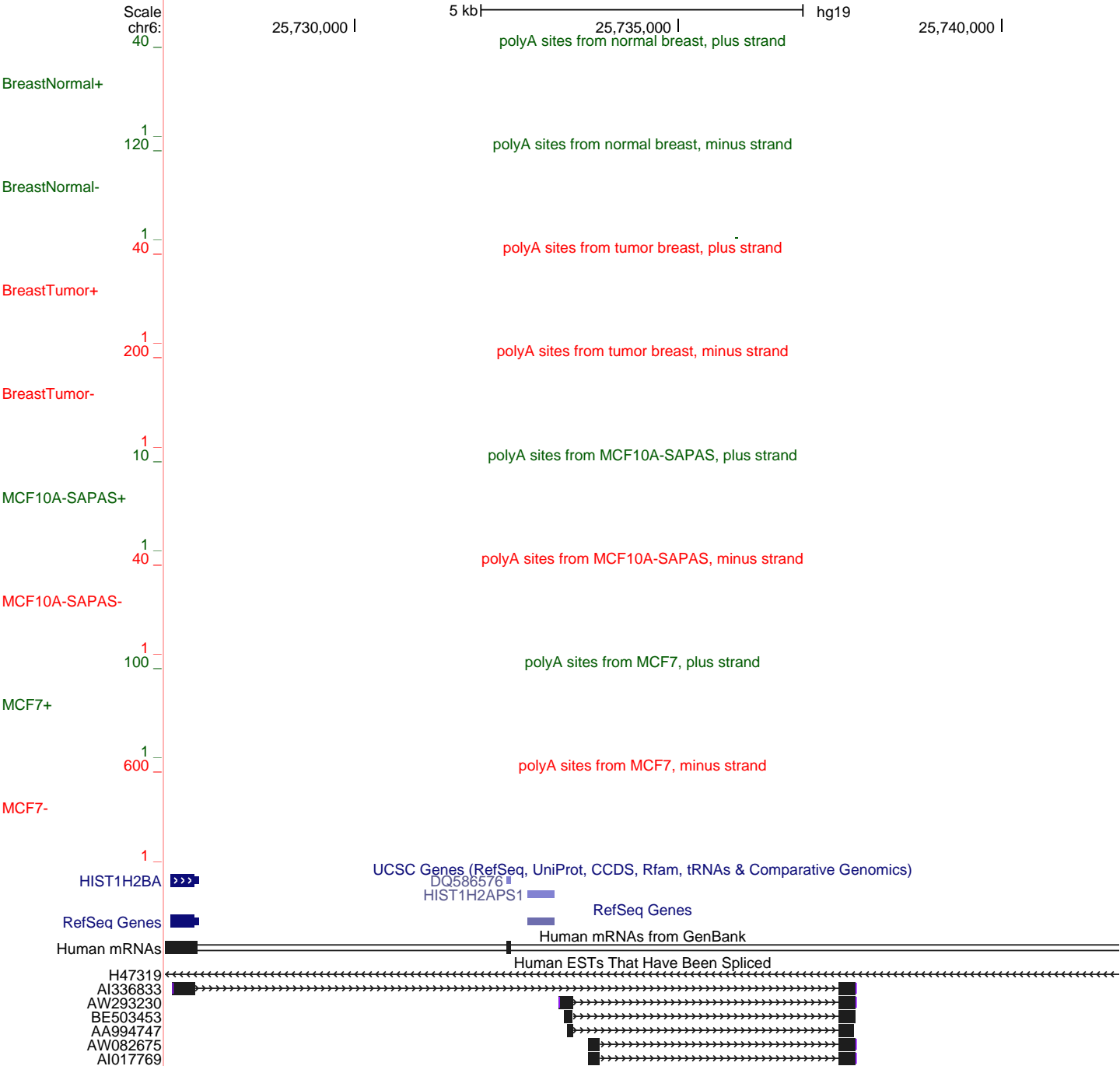

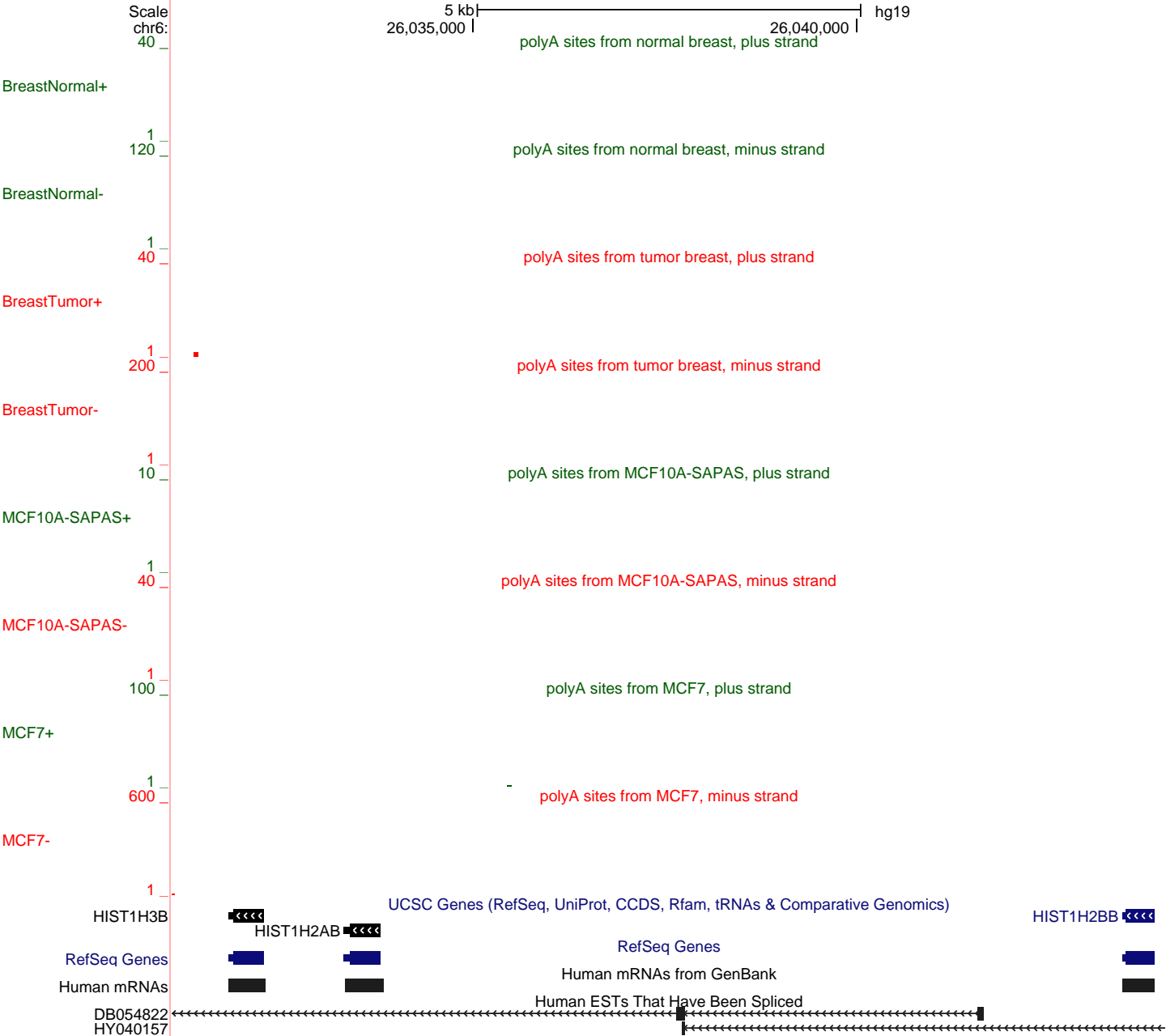

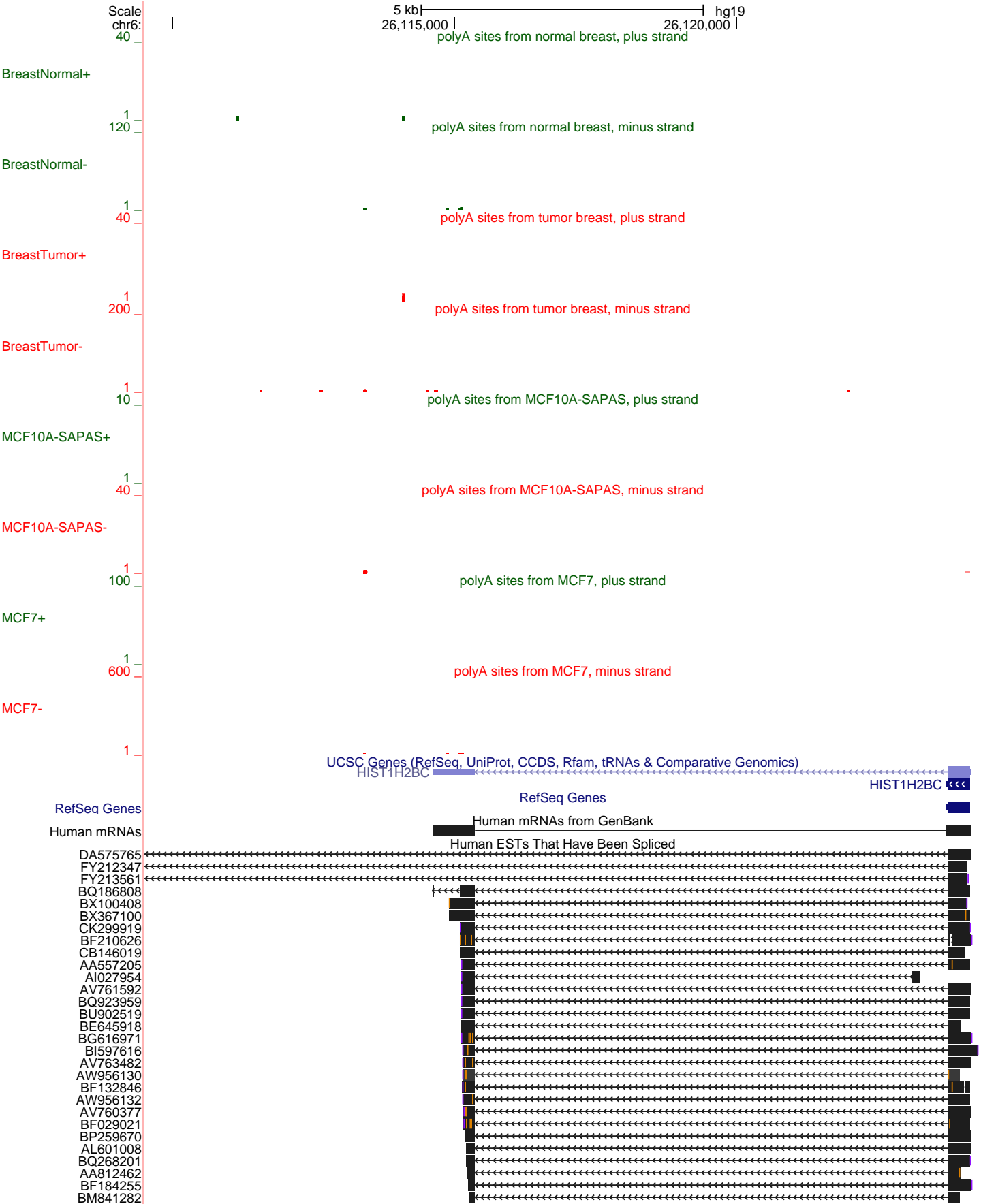

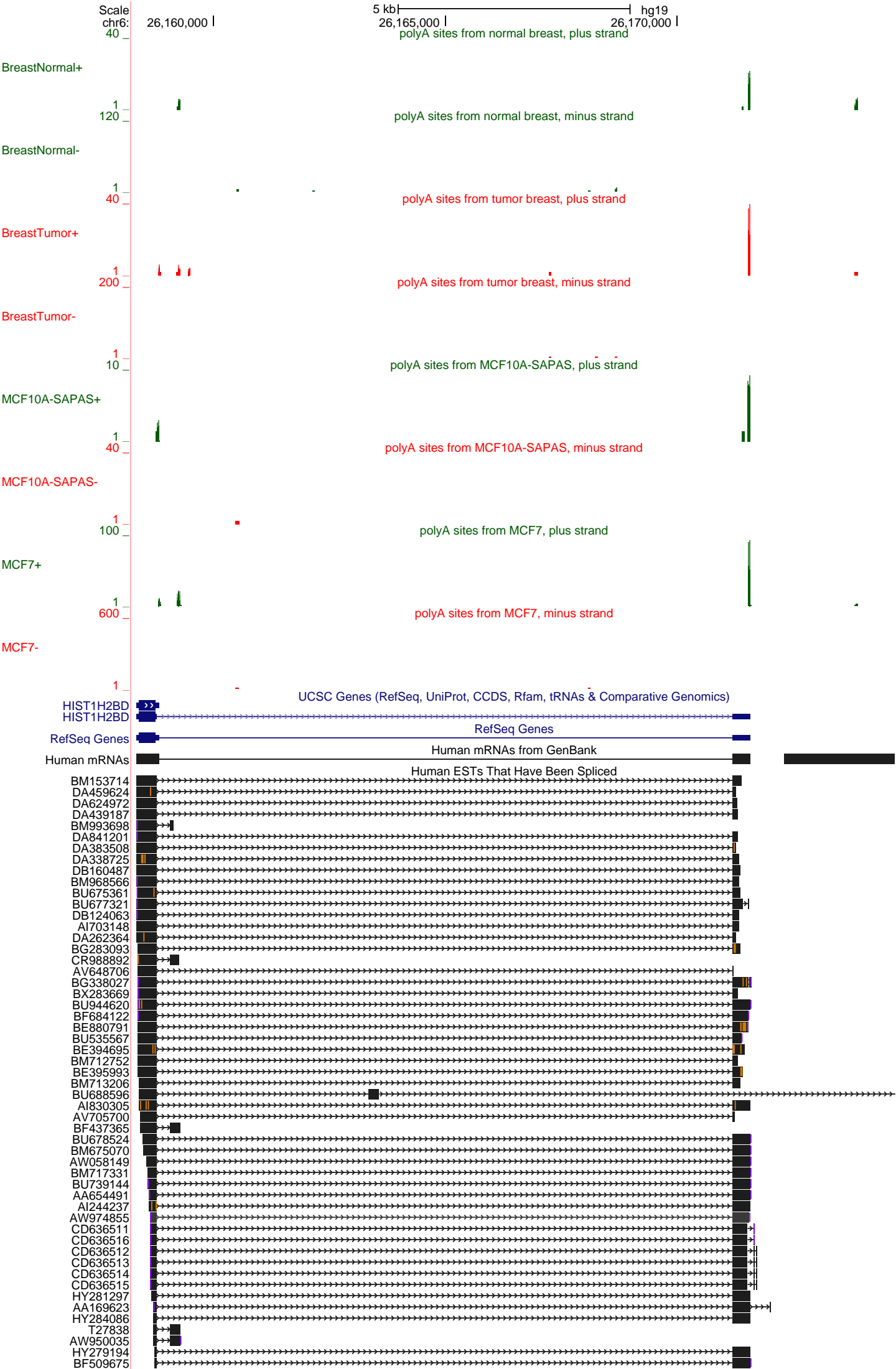

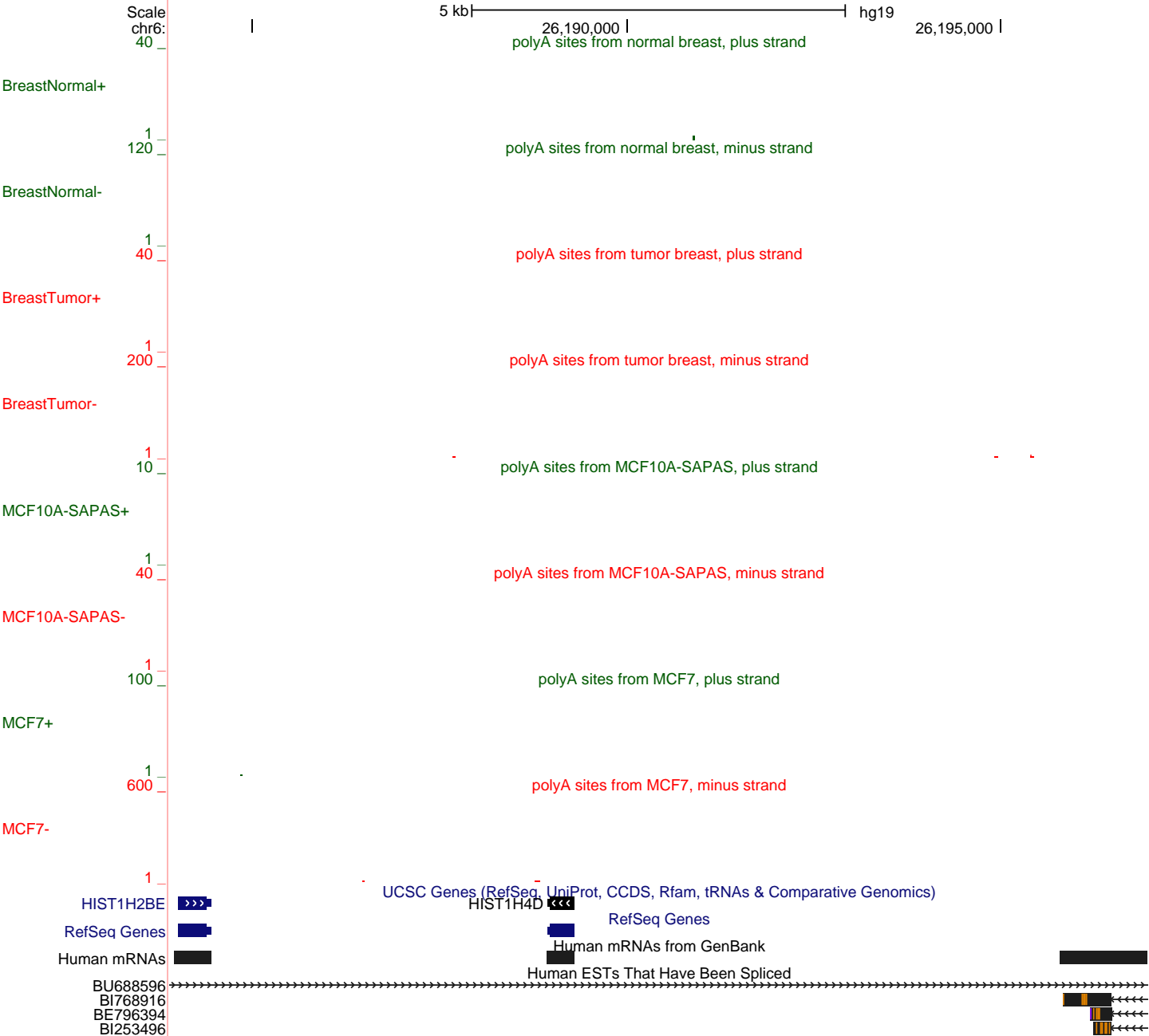

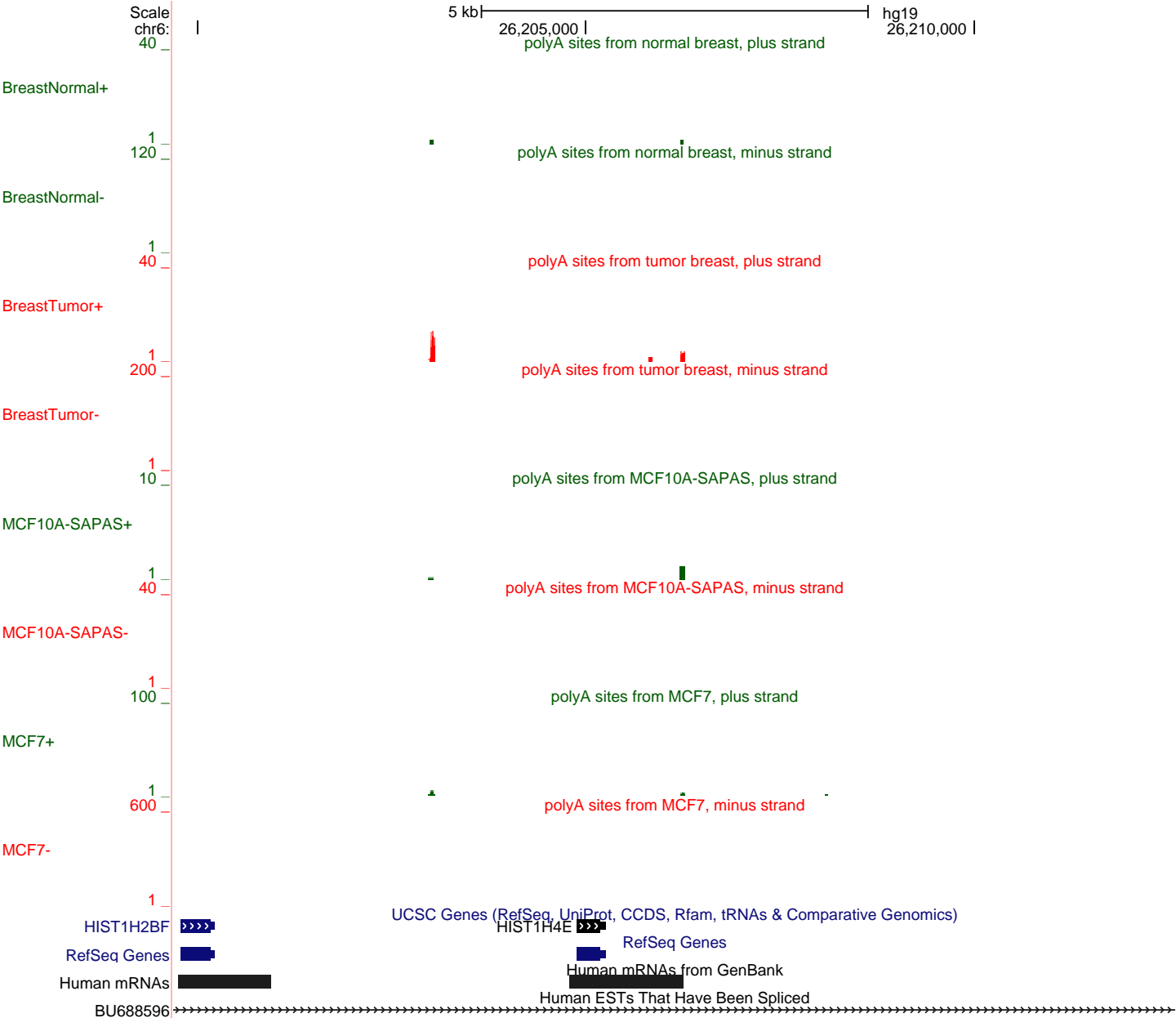

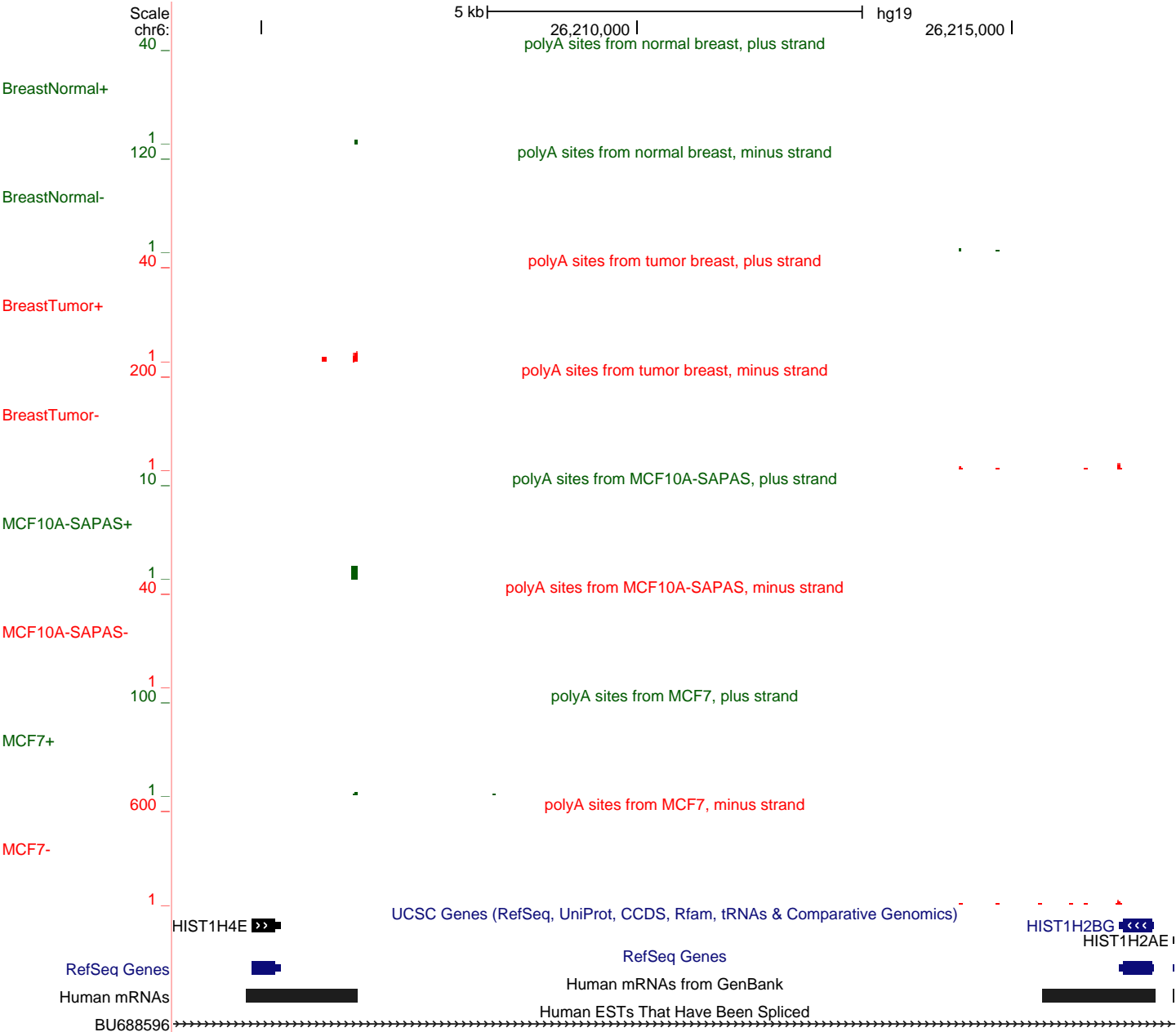

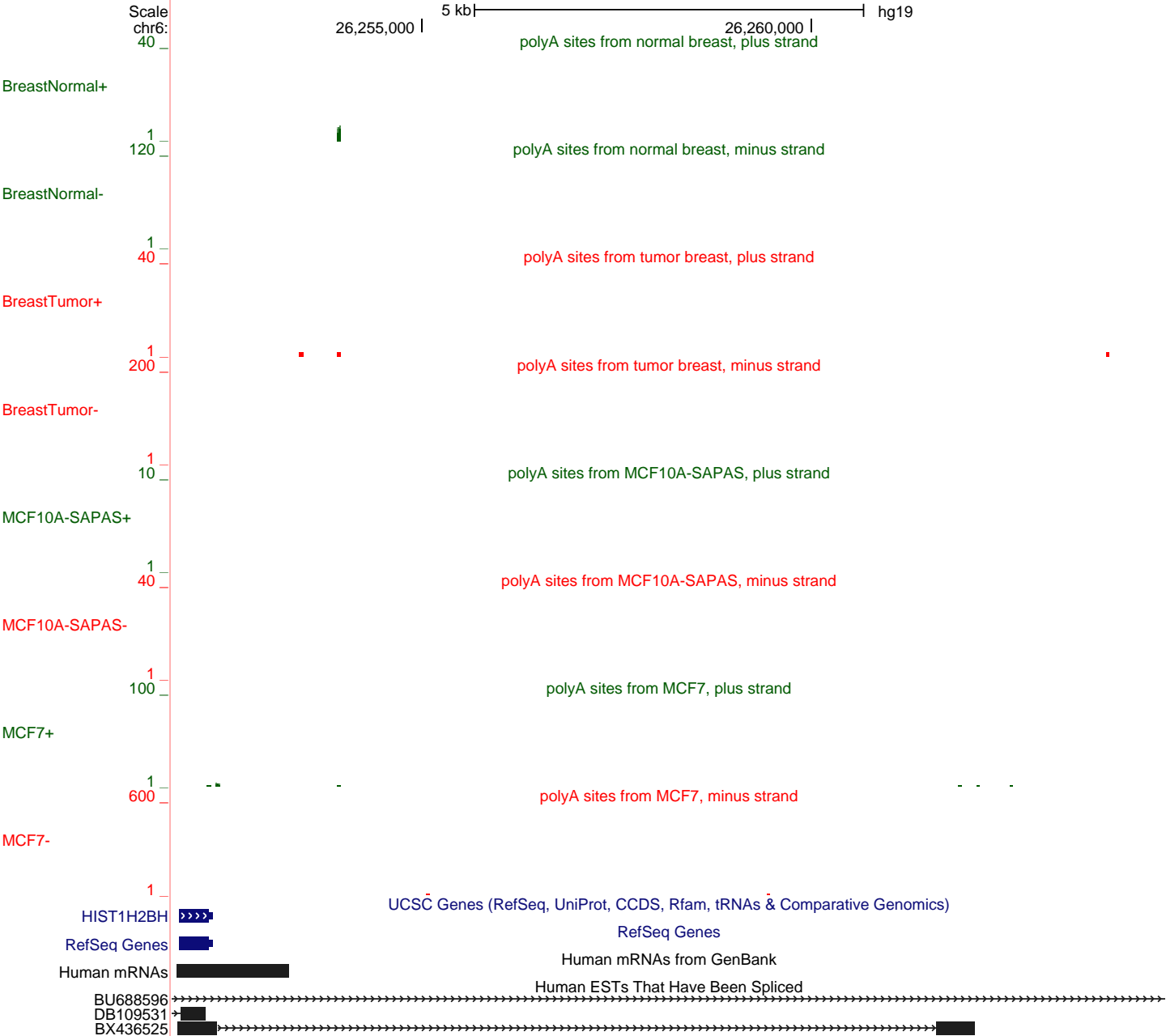

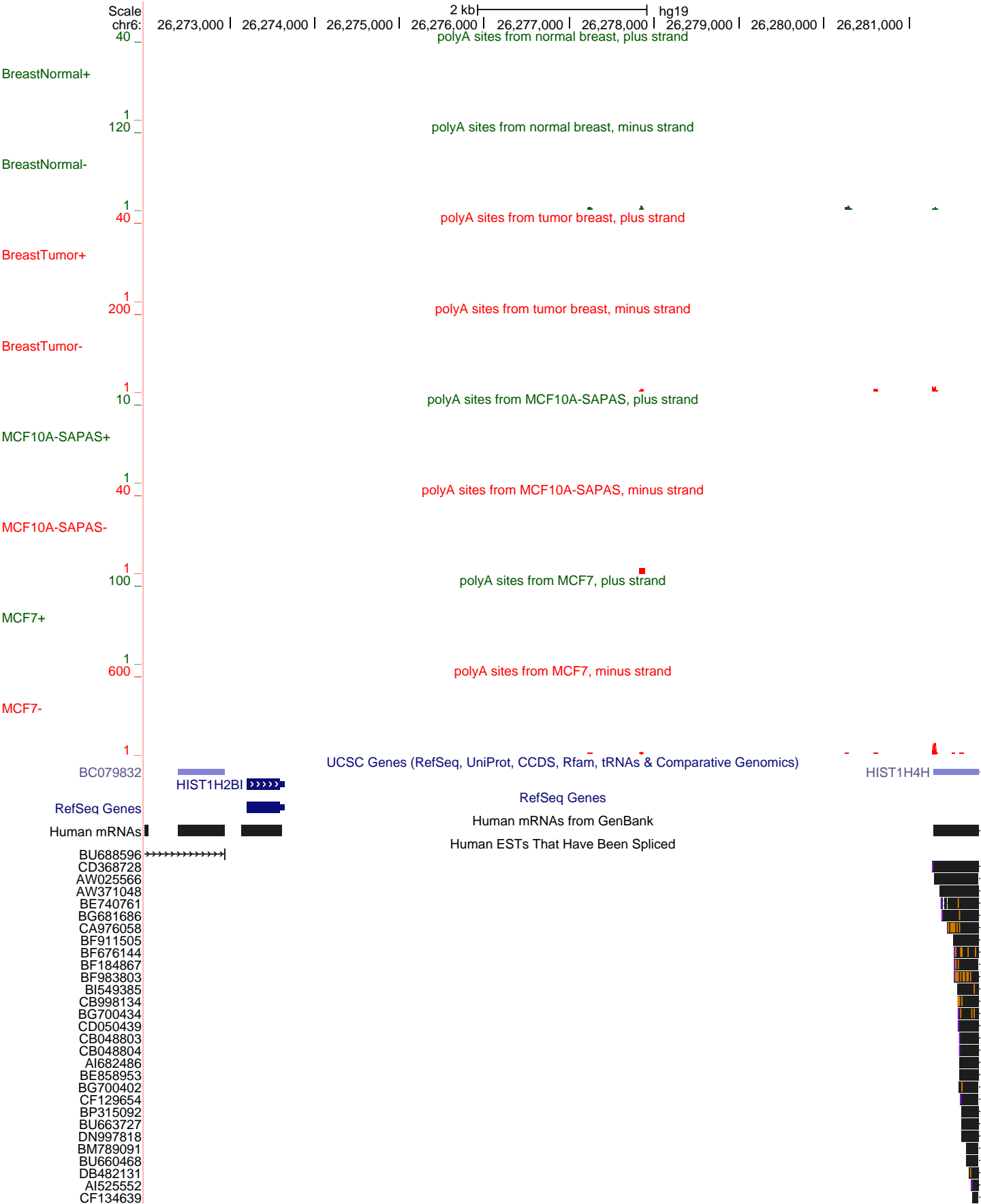

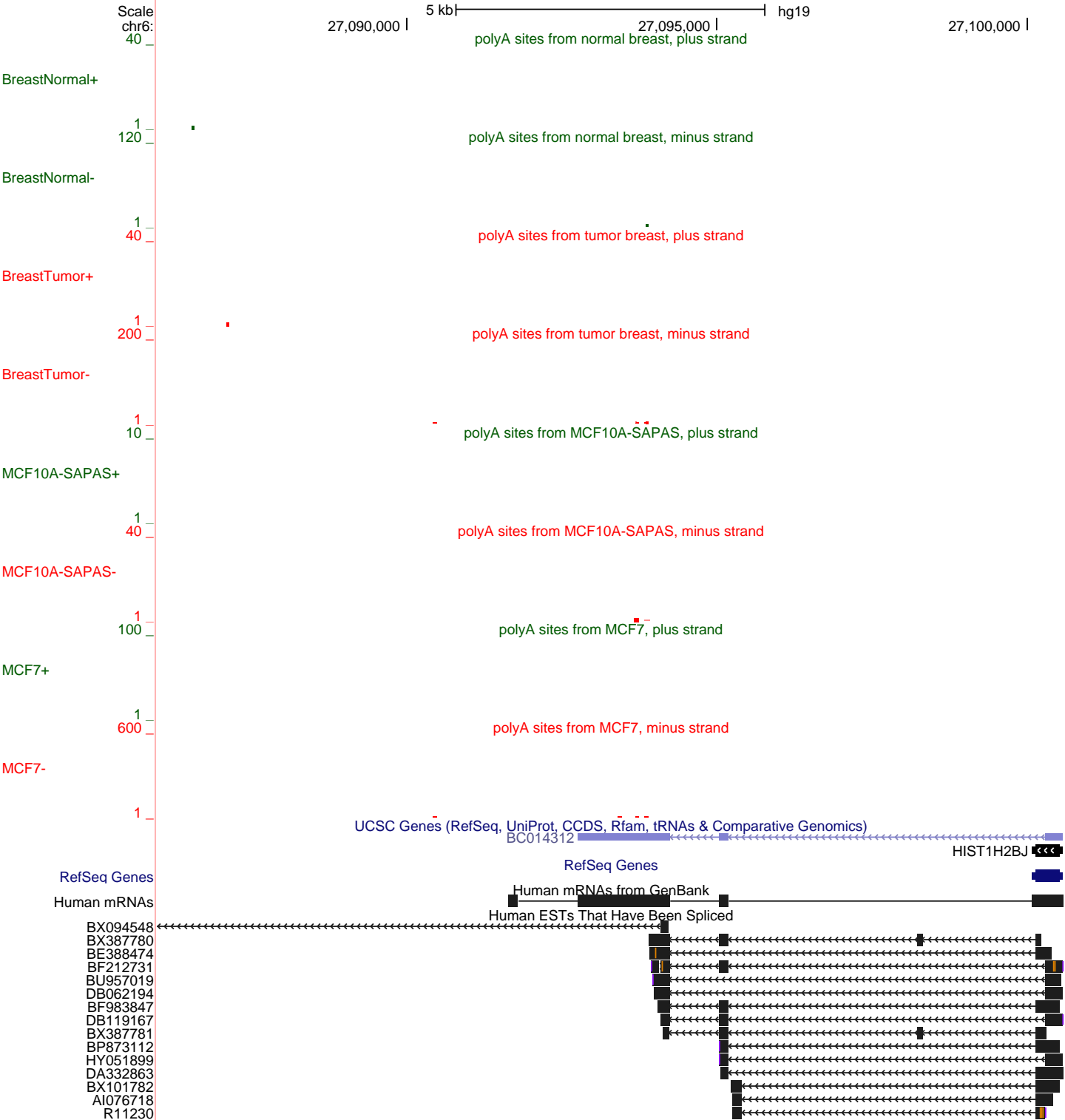

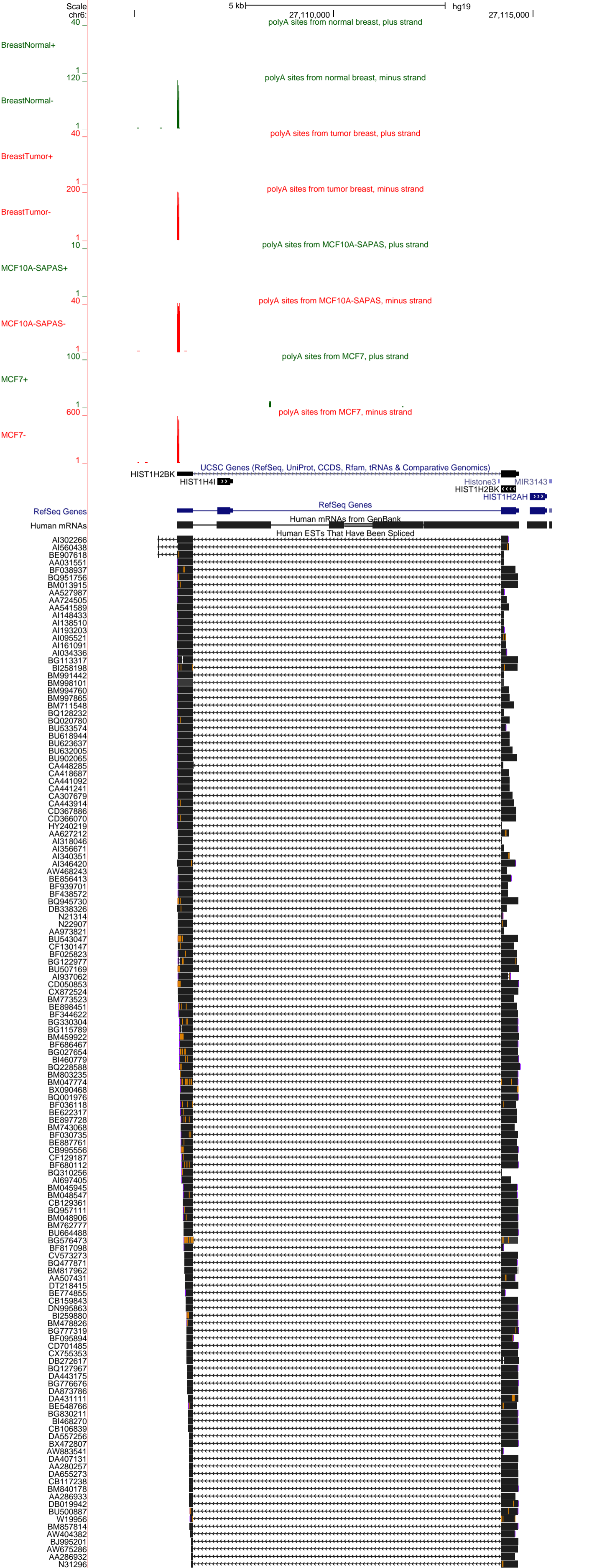

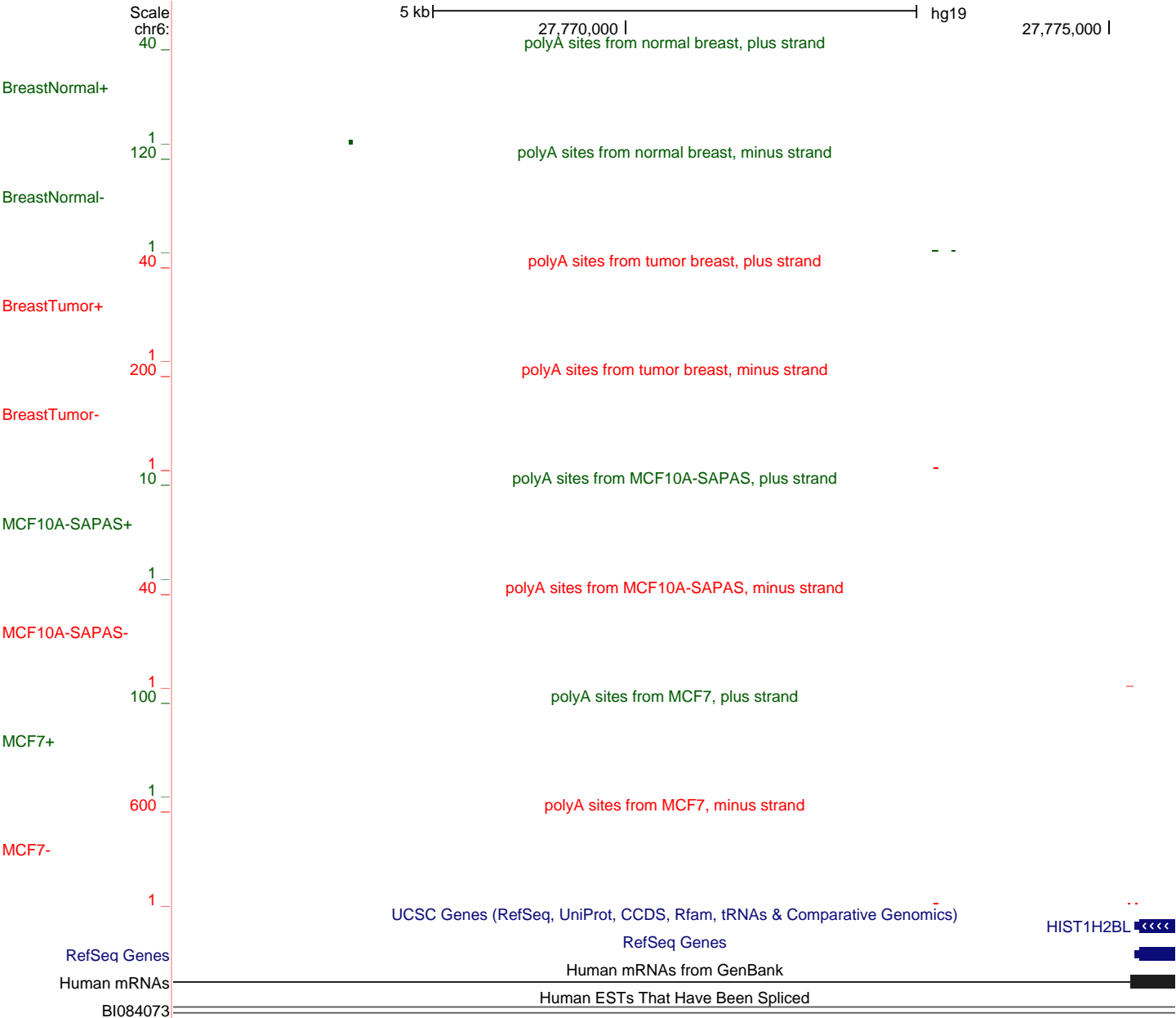

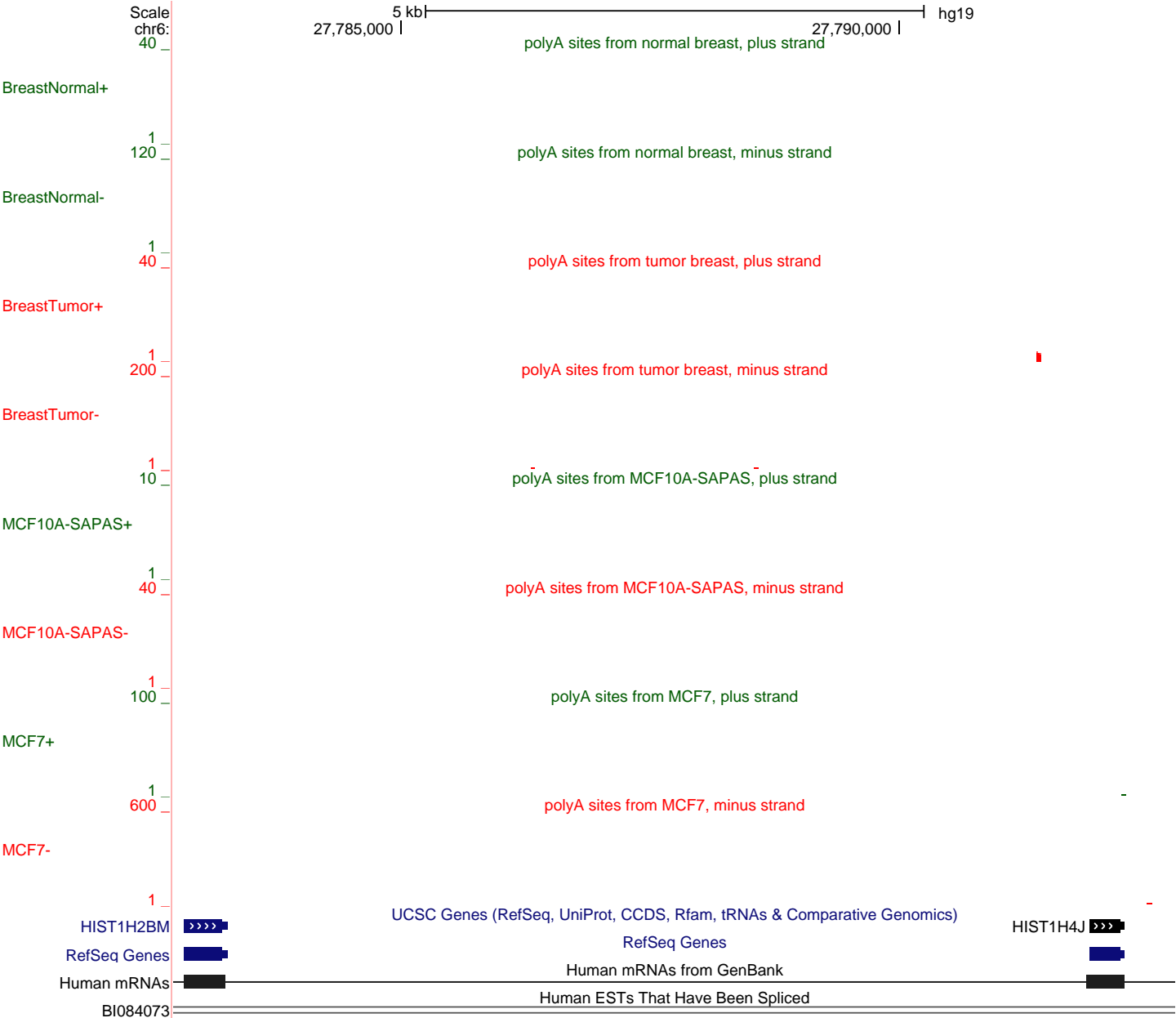

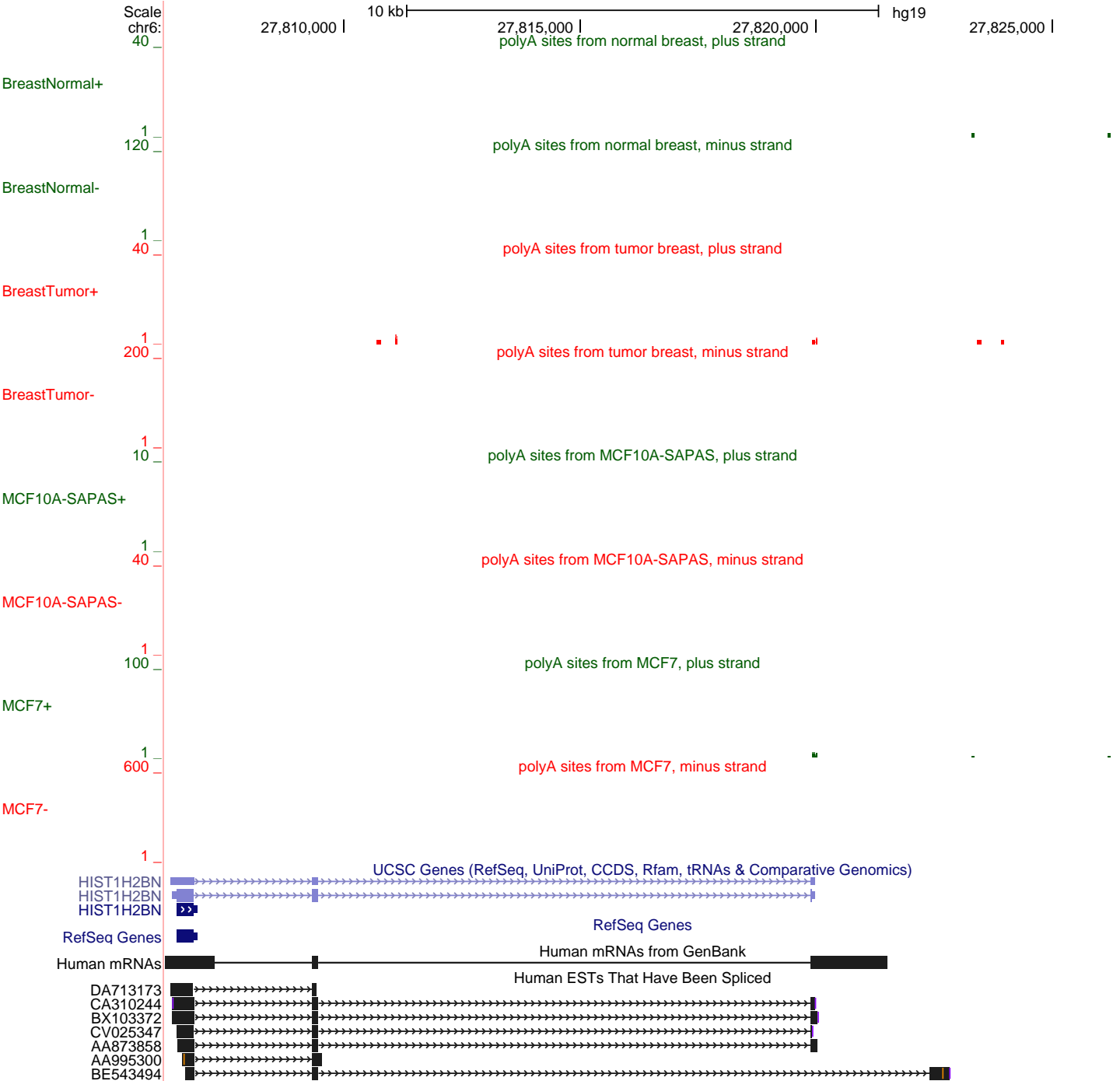

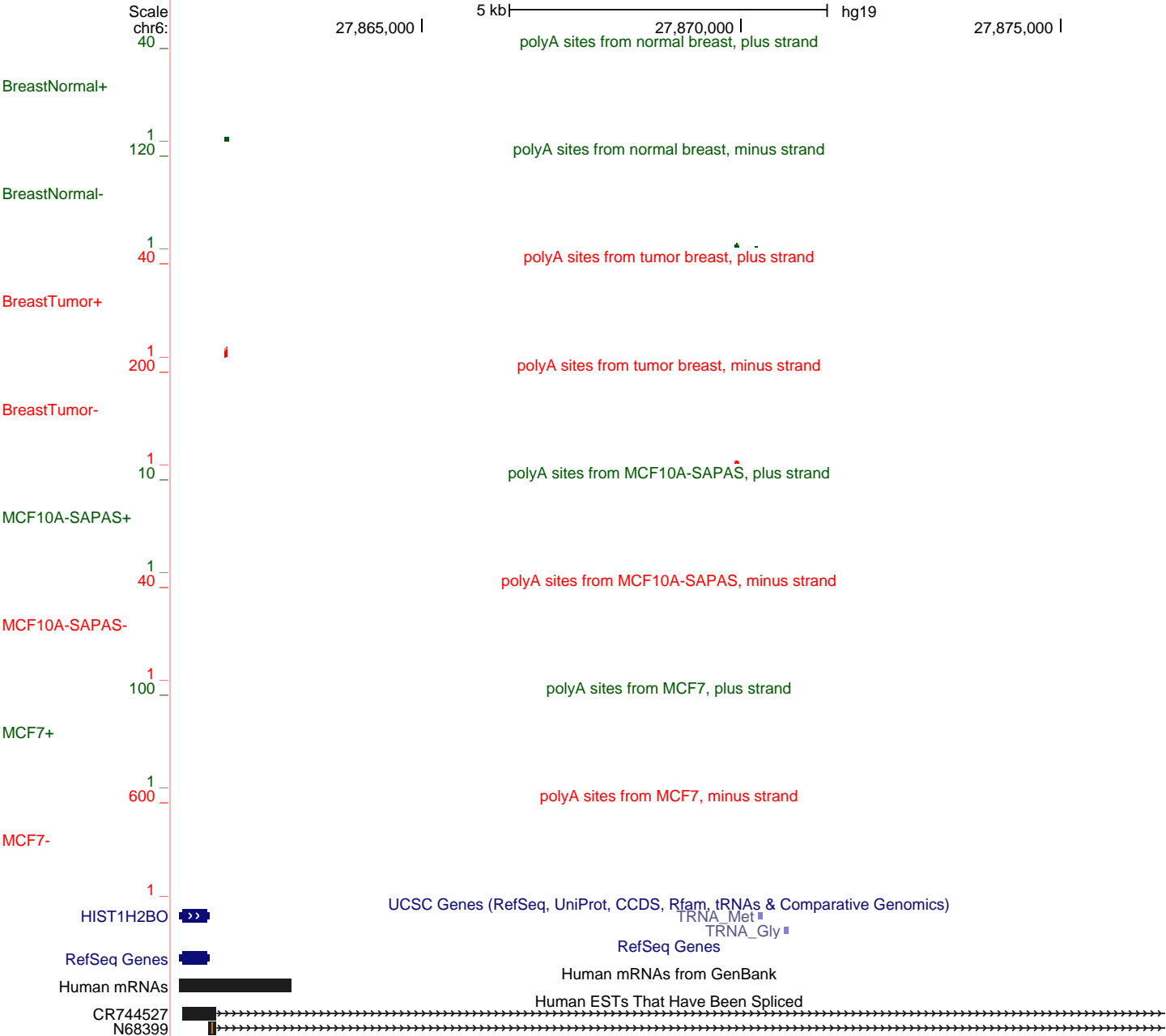

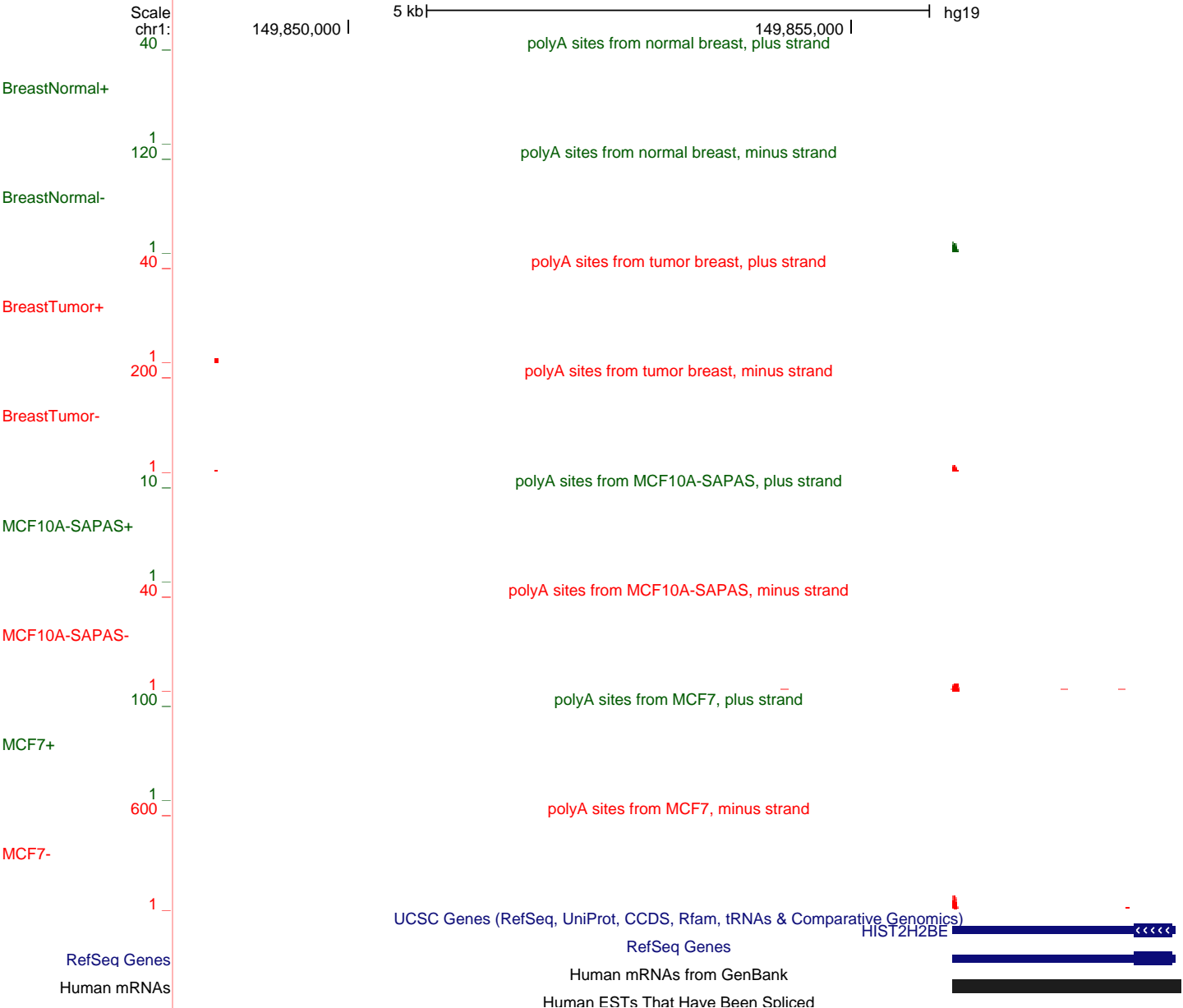

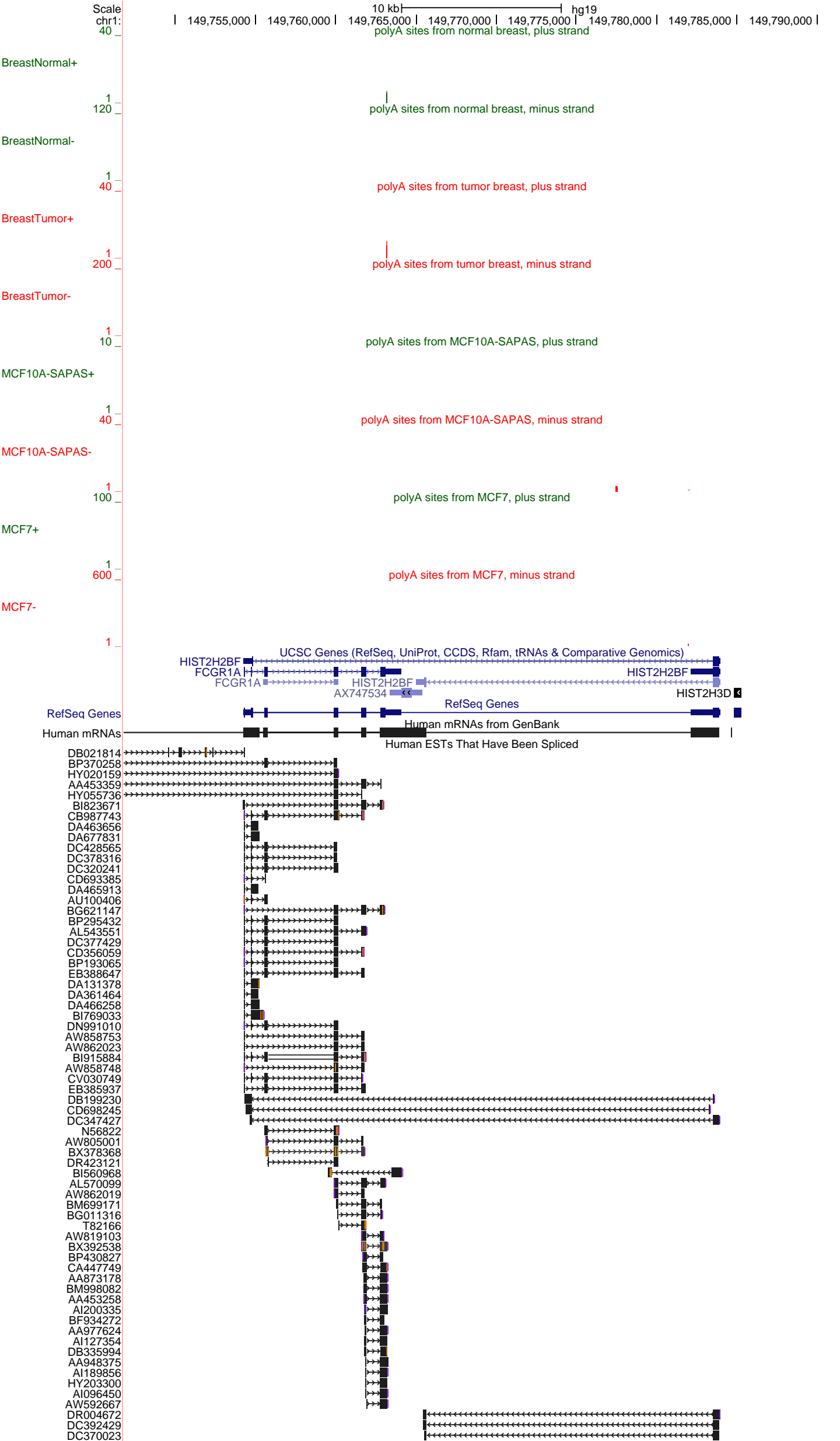

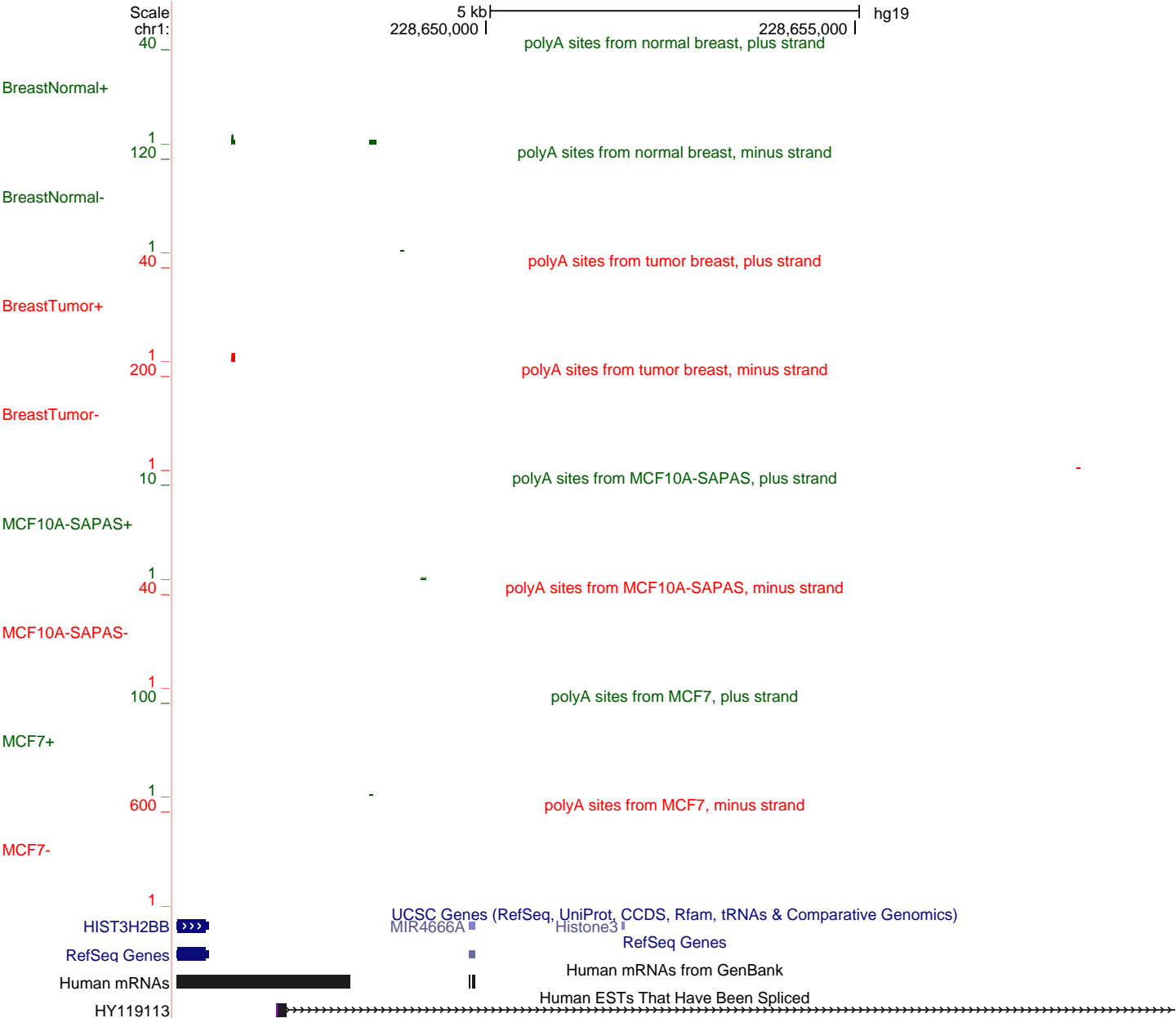

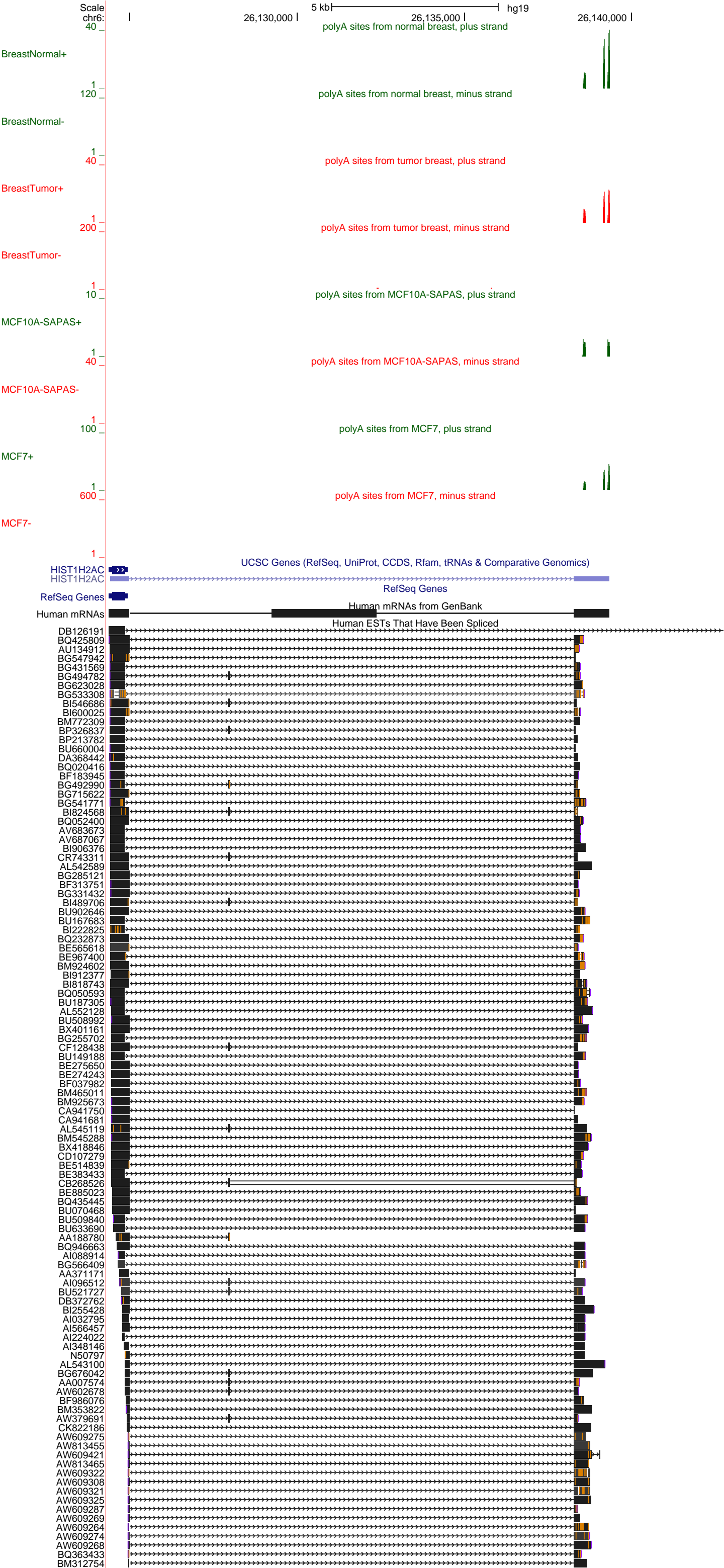

Supplement: Figure S3 — UCSC genome browser views of DRS data depicted in Fig. 3 . (PDF) [file pone.0063745.s003.pdf]
