# Supplementary material for: A Subset of Histone H2B Genes Produces Polyadenylated mRNAs under a Variety of Cellular Conditions
Source: PLoS One. 2013 May 22;8(5):e63745. doi: 10.1371/journal.pone.0063745 (PMC3661734; doi:10.1371/journal.pone.0063745)
Supplement: Table S1 — Primers used in this study. (DOCX) [file pone.0063745.s004.docx]

Supplementary Table S1

Primers used in this study

| **Name** | **Sequence (5’-3’)** | **Reference (if present)** |
| --- | --- | --- |
| ALPL F | TGGGCCAAGGACGCTGGGAA | (Karpiuk, 2012) |
| ALPL R | AAGGCCTCAGGGGGCATCTCG | (Karpiuk, 2012) |
| BGLAP F | GCCCTCACACTCCTCGCCCT | (Karpiuk, 2012) |
| BGLAP R | CGGGTAGGGGACTGGGGCTC | (Karpiuk, 2012) |
| HIST1H2BA 145 F | CAGGTCCATCCGGACACTGGCA | This study |
| HIST1H2BA 246 R | CAAACGTGATGCCTCGCTCGCT | This study |
| HIST1H2BB 151 F | CCCGACACCGGCATCTCATCCA | This study |
| HIST1H2BB 352 R | CCTTAGTGCCCTCGGACACAGCA | This study |
| HIST1H2BC 47 F | AGAAGGCAGTGACCAAAGCGCAG | This study |
| HIST1H2BC 183 R | GCCCATGGCCTTGGAAGAGATGC | This study |
| HIST1H2BD 47 F | ACGATGCCTGAACCTACCAA | This study |
| HIST1H2BD 115 R | AGCCTTAGTCACCGCCTTCT | This study |
| HIST1H2BE 55 F | GTGACCAAGGCGCAGAAGAAGGAC | This study |
| HIST1H2BE 174 R | TTTAGAGGAGATGCCGGTGTCGGG | This study |
| HIST1H2BF 157 F | ACCGGCATCTCATCCAAGGCCA | This study |
| HIST1H2BF 340 R | TGACACGGCGTGCTTAGCCAG | This study |
| HIST1H2BG 84 F | AGAAGCGCAAGCGCAGTCGT | This study |
| HIST1H2BG 252 R | TAGTGGGCCAGACGGGAAGCC | This study |
| HIST1H2BH 87 F | GCGTAAACGCAGCCGCAAGG | This study |
| HIST1H2BH 323 R | GCCAGTTCCCCAGGCAGCAG | This study |
| HIST1H2BI 278 F | GGGAGATCCAAACGGCTGTGCG | This study |
| HIST1H2BI 421 R | GAGCCTTTGGGTCGTTAGCGCTTT | This study |
| HIST1H2BJ 55 F | GCCAGCGAAGTCTGCTCCCG | This study |
| HIST1H2BJ 156 R | CTCTCCTTGCGGCTGCGCTT | This study |
| HIST1H2BK 8F | TGCTGCTCGTCTCAGGCTCGT | This study |
| HIST1H2BK 152 R | CTCTCCTTGCGGCTGCGCTT | This study |
| HIST1H2BL 69 F | CCAAGAAGGCGGTGACCAAGGC | This study |
| HIST1H2BL 196 R | AGAAGAGATGCCGGTGTCGGGG | This study |
| HIST1H2BM 291 F | GGCCGTGCGCCTACTGCTAC | This study |
| HIST1H2BM 320 R | GGTGTGGGTCACGGCGGAAC | This study |
| HIST1H2BN 61F | CAAAGTCCGCTCCTGCCCCG | This study |
| HIST1H2BN 162R | TGACCGAACGTTCCGCGGTG | This study |
| HIST1H2BO 23 F | TTCACTCTCCTCCGCCATGCCC | This study |
| HIST1H2BO 146 R | CTCTTTGCGGCTGCGCTTGC | This study |
| HIST2H2BE 767 F | CCTGGTGGCTCCTTGGGTCTGT | This study |
| HIST2H2BE 958 R | TATCCACAGGAGGCCCCATCGC | This study |
| HIST2H2BF 241 F | CCTCCACCCCACCACCCCTC | This study |
| HIST2H2BF 397 R | ATGGACTCGGGAACCGCCGA | This study |
| HIST3H2BB 232 F | TCTTCGAGCGCATCGCCAGC | This study |
| HIST3H2BB 423 R | CAGGACGCCGAGGAACGCC | This study |
| HIST1H2BD PolyA F | CCAACTCATCCTGGTTTGCT | (Pirngruber *et al.*, 2009) |
| HIST1H2BD PolyA R | TCCCCTCGGTAACCTTCTTT | (Pirngruber *et al.*, 2009) |
| HIST1H2AC Total F | GACGAGGAGCTCAACAAACTG | (Pirngruber *et al.*, 2009) |
| HIST1H2AC Total R | ACCTGTCAAATCACTTGCCC | (Pirngruber *et al.*, 2009) |
| HIST1H2AC PolyA F | CCTGTCCACTGTTGGTAGGC | (Pirngruber *et al.*, 2009) |
| HIST1H2AC PolyA R | TTCACTTACCACCATTCCAGC | (Pirngruber *et al.*, 2009) |
| HIST1H2BD Spl 402 F | CCGTCACCAAGTACACCAGTT | This study |
| HIST1H2BD Spl 614 R | TCCCCTCGGTAACCTTCTTT | This study |
| HIST1H2AC Spl 505F | CCCCTACCGTTTCAAAGGA | This study |
| HIST1H2AC Spl 632R | ATTGGTAAGTTTGGCAGGCA | This study |
| HNRNPK F | ATCCGCCCCTGAACGCCCAT | (Karpiuk, 2012) |
| HNRNPK R | ACATACCGCTCGGGGCCACT | (Karpiuk, 2012) |
| PPARG F | ACCTCCGGGCCCTGGCAAAA | (Karpiuk, 2012) |
| PPARG R | TGCTCTGCTCCTGCAGGGGG | (Karpiuk, 2012) |
| RPLP0 F | GATTGGCTACCCAACTGTTG | (Fritah et al., 2005) |
| RPLP0 R | CAGGGGCAGCAGCCACAAA | (Fritah et al., 2005) |
| rRNA ITS1/5.8S F | GGCCTGAGGCAACCCCCTCT | This study |
| rRNA ITS1/5.8S R | GACGCACGAGCCGAGTGATCC | This study |
| rRNA 5.8S F | GCGGTGGATCACTCGGCTCG | This study |
| rRNA 5.8S R | CGTAGCCCCGGGAGGAACCC | This study |
| h18S rRNA F | AACTGAGGCCATGATTAAGA | This study |
| h18S rRNA R | GGAACTACGACGGTATCTGA | This study |
